# Neuromuscular junction pathology is correlated with differential motor unit vulnerability in spinal and bulbar muscular atrophy

**DOI:** 10.1186/s40478-022-01402-y

**Published:** 2022-07-05

**Authors:** Elana Molotsky, Yuhong Liu, Andrew P. Lieberman, Diane E. Merry

**Affiliations:** 1 Department of Biochemistry and Molecular Biology, Sidney Kimmel Medical College, Thomas Jefferson University, Jefferson Alumni Hall, Rm. 411E, Philadelphia, PA 19107 USA; 2 Department of Pathology, University of Michigan Medical School, Ann Arbor, MI USA

**Keywords:** SBMA, Neuromuscular junction, Skeletal muscle, Motor neuron

## Abstract

Spinal and bulbar muscular atrophy (SBMA) is an X-linked, neuromuscular neurodegenerative disease for which there is no cure. The disease is characterized by a selective decrease in fast-muscle power (e.g., tongue pressure, grip strength) accompanied by a selective loss of fast-twitch muscle fibers. However, the relationship between neuromuscular junction (NMJ) pathology and fast-twitch motor unit vulnerability has yet to be explored. In this study, we used a cross-model comparison of two mouse models of SBMA to evaluate neuromuscular junction pathology, glycolytic-to-oxidative fiber-type switching, and cytoskeletal alterations in pre- and postsynaptic termini of tibialis anterior (TA), gastrocnemius, and soleus hindlimb muscles. We observed significantly increased NMJ and myofiber pathology in fast-twitch, glycolytic motor units of the TA and gastrocnemius compared to slow-twitch, oxidative motor units of the soleus, as seen by decreased pre- and post-synaptic membrane area, decreased pre- and post-synaptic membrane colocalization, increased acetylcholine receptor compactness, a decrease in endplate area and complexity, and deficits in neurofilament heavy chain. Our data also show evidence for metabolic dysregulation and myofiber atrophy that correlate with severity of NMJ pathology. We propose a model in which the dynamic communicative relationship between the motor neuron and muscle, along with the developmental subtype of the muscle, promotes motor unit subtype specific vulnerability, metabolic alterations, and NMJ pathology.

**Supplementary Information:**

The online version contains supplementary material available at 10.1186/s40478-022-01402-y.

## Background

Spinal and bulbar muscular atrophy (SBMA; Kennedy’s Disease) is an X-linked neuromuscular neurodegenerative disease caused by a polyglutamine-encoding CAG repeat expansion in exon 1 of the androgen receptor (*Ar*) gene [13, 30]. SBMA is characterized by limb and bulbar muscle weakness and atrophy and by the loss of lower motor neurons from the spinal cord and brainstem motor nuclei. Mutant AR-containing intranuclear inclusions are observed in remaining neurons, muscle, and to a lesser extent in tissues throughout the body that show little or no degenerative phenotype. The combination of lower motor neuron loss and muscle atrophy results in symptoms such as fasciculations, dysarthria, dysphagia, muscle weakness, and reduced muscle contractility [2]. Symptoms typically present between 30 and 50 years of age, and bulbar degeneration predisposes patients to potentially fatal aspiration pneumonia [17, 64]. Due to the requirement for testosterone for disease progression, SBMA is male-specific, although female patients are known to show mild or subclinical symptoms [65]. Currently there is no cure for this disease.

SBMA was historically classified as a lower motor neuron disease, and in SBMA patients, the loss of lower motor neurons results in a decrease in the number of functioning motor units compared to age-matched controls [67]. Mouse models recapitulate many aspects of motor neuron pathology such as intranuclear inclusions containing polyglutamine-expanded AR, electrophysiological alterations, neuronal loss, and decreased somal unphosphorylated neurofilament heavy chain [5, 9, 26, 71]. While earlier studies focused on the role of neurogenic degeneration of the motor unit in SBMA pathogenesis, many recent studies have elucidated a primary role for myogenic atrophy in SBMA. For example, when CAG-expanded *Ar* is selectively silenced in muscles of SBMA mouse models, disease phenotypes are substantially rescued, providing evidence for a primary myopathic disease mechanism [9, 34]. Moreover, metabolic and transcriptional dysregulation in muscle fibers could have non-cell autonomous effects on the health of the innervating motor neuron [47, 58], potentially contributing to motor neuron pathology in SBMA and degeneration of the motor unit.

Evidence of both myogenic and neurogenic disease processes places the neuromuscular junction (NMJ), the chemical synapse of the motor unit, at a focal point in SBMA pathogenesis. The unique synaptic structure of the NMJ ensures a high safety factor for neuromuscular transmission. Axon terminals of a pre-synaptic motor neuron must completely overlap with the pretzel-shaped acetylcholine receptor (AChR) clusters on the postsynaptic endplate to ensure that an action potential in the motor neuron reliably promotes contraction of the muscle fiber [31]. NMJs in multiple neuromuscular disease states including amyotrophic lateral sclerosis (ALS), spinal muscular atrophy (SMA), and SBMA show a variety of structural pathologies such as fragmented AChR clusters, lack of AChR colocalization with the presynaptic terminal, denervation, loss of cytoskeletal elements, and a change in size or complexity (i.e., branching pattern) [36, 43, 51, 60]. Previous studies in SBMA mouse models revealed significant changes in NMJ structure and function [7, 27, 51, 60, 72]; however, the specificity of NMJ pathology to muscle and motor unit subtype has yet to be established.

Motor units, defined as the lower motor neuron and the muscle fibers it innervates, are classified as slow-twitch oxidative (Type I), fast-twitch oxidative (Type IIa), or fast-twitch glycolytic (Type IIb/x) based on the contractile properties of the muscular cytoskeleton. ‘Oxidative’ and ‘glycolytic’ refer to the metabolic machinery used to create ATP in the specified fiber type. Glycolytic (fast-twitch fibers) rely on glycolysis to provide energy, while oxidative (generally slow-twitch fibers) use both glycolysis and oxidative phosphorylation to create a sustained source of ATP for prolonged contraction. Importantly, the contractile properties of the muscle fiber are determined by the firing rate of its innervating motor neuron, which promotes a transcriptional program specific to muscle fiber types [66]. Preferential loss of fast-twitch motor units has been seen in other neurodegenerative diseases such as ALS [11, 18, 19, 54], and glycolytic-to-oxidative fiber-type switching has been seen in mouse models of SBMA [7, 55, 58], as well as during normal aging [25]. In SBMA patients, decreased tongue pressure and grip strength, along with fast-twitch muscle fiber atrophy, suggest selective fast motor unit degeneration [17, 23, 73]; however, the vulnerability of fast-twitch motor units, and the relationship between NMJ structural pathology and the differential vulnerability of fast- and slow-twitch motor units in SBMA has yet to be evaluated.

In this study, we aimed to evaluate the NMJ pathology in relation to fiber type vulnerability to further understand the intersection of neuronal and myofibrillar pathology in multiple muscles of SBMA mouse models. We determined the relationship between NMJ structural pathology and motor unit subtype (fast- vs. slow-twitch) using two mouse models of SBMA—a transgenic, CNS expression-focused mouse model of SBMA [5] and a knock-in mouse model of SBMA [74, 75]. We evaluated multiple aspects of NMJ morphology in three muscles of distinct fiber type—gastrocnemius and tibialis anterior (TA), both largely fast-twitch, glycolytic muscles, and soleus, a largely slow-twitch, oxidative muscle. We hypothesized that NMJ structural pathology in these mouse models would correlate with the increased vulnerability of fast-twitch motor units seen in both SBMA mouse models and patients, showing, for example, increased endplate fragmentation and decreased pre- and post-synaptic colocalization.

We observed significant NMJ structural pathology in all three muscles of both mouse models, with more severe NMJ pathology in muscles composed primarily of fast-twitch myofibers. Cytoskeletal alterations in both motor neurons and muscle, namely decreased NFH and altered myosin heavy chain expression, provide evidence for both neuropathic and myopathic contributions to NMJ pathology in these SBMA models. Analysis of muscle histology to further understand myofiber changes in relation to NMJ pathology revealed a prominent shift to oxidative metabolism and a decrease in cross sectional area in all muscles of both mouse models, with fast-twitch muscles showing more severe muscle pathology. These quantitative studies provide significant evidence of correlated neuronal and muscular dysregulation that contribute to NMJ pathology, which is more severe in fast-twitch motor units. We propose a model in which both the contractile properties of the motor unit and the developmental subtype of the muscle contribute to disease pathology at the NMJ of SBMA model mice and highlight the importance of robust and quantitative NMJ evaluations for the understanding these contributions.

## Methods

### Mouse models

Animal care and experimental procedures were conducted in accordance with the Thomas Jefferson University Institutional Animal Care and Use Committee (IACUC). Transgenic Model: The transgenic model, previously described in Chevalier-Larsen et al., 2004 [5], uses the prion protein promoter to express an expanded human AR containing 112 CAG repeats (+ 1 CAA codon, encoding a total of 113 glutamine residues within the human AR protein) primarily in the central nervous system. Knock-in Model: The knock-in model, previously described in Yu et al., [74, 75], replaces part of the exon 1 of the mouse androgen receptor gene with a portion of human *Ar* exon 1 containing 112 CAG repeats (+ 1 CAA codon, encoding a total of 113 glutamine residues within the human AR protein) resulting in endogenous expression levels and pattern of polyglutamine expanded androgen receptor.

### Neuromuscular junction analysis

Muscles of 3 mice of each genotype (1-year-old non-transgenic and transgenic mice, and 6-month-old wildtype and knock-in mice) were dissected. Gastrocnemius, tibialis anterior, and soleus muscles were dissected from SBMA mice and littermate controls and immediately placed into 4% paraformaldehyde (PFA) for 30 min. Muscles were then teased into small bundles under a dissecting microscope in PBS and stained according to Martin et al., 2015 [41]. Briefly, teased muscle was incubated in 0.1 M glycine solution, incubated in AlexaFluor-647-conjugated α-bungarotoxin, then incubated in pre-cooled methanol. Muscles were incubated in blocking buffer (2% bovine serum albumin in 0.2% Triton-X-100) and then incubated with primary and then secondary antibodies in blocking buffer. Muscles were mounted on microscope slides using Fluoromount-G mounting medium and imaged on a Nikon A1R confocal microscope. At least 20 neuromuscular junctions from each muscle were imaged. Antibodies used are provided in supplementary methods.

### Image analysis of neuromuscular junctions

At least 20 neuromuscular junctions from each muscle were evaluated with the following protocol using ImageJ, adapted from Jones et al. [24]. Full analysis protocol is provided in supplementary methods (schematic shown in Additional File 1: Fig. S1).

### Immunohistochemical analysis of muscle

NADH-TR (diaphorase): Muscle sections were incubated in NADH-tetrazolium solution (NADH, Sigma N8129; Nitro blue tetrazolium, Sigma N6876) for 30 min at 37 °C. Unbound solution was removed from muscle sections using by successive washes with 30% acetone, 60% acetone, 90% acetone, 60% acetone, and 30% acetone. Sections were mounted with CitriSolve. Images were taken at 10X using an EVOS M7000 microscope. Intensity of NADH staining and cross-sectional area were analyzed in ImageJ by manually selecting each muscle fiber and recording mean intensity and area. 3 images were analyzed ($\ge$ 574 fibers) per muscle for each genotype.

Androgen Receptor: Muscle sections of gastrocnemius from one knock-in mouse and one transgenic mouse were fixed in 4% PFA for 20 min, and stained as in Montie et al. [46]. Images were taken at consistent exposure on an EVOS M7000 microscope at 60x. 15 images per muscle were evaluated in ImageJ by selecting a region of interest for each muscle fiber and measuring mean intensity of AR fluorescence. All images were taken at the same exposure.

### Western blot analysis

Gastrocnemius, tibialis anterior, and soleus muscles were dissected from 3 mice of each genotype and flash-frozen in liquid nitrogen (12-month-old non-transgenic and transgenic, 9 month old knock-in mice). Muscles were manually pulverized with a mortar and pestle and added to lysis buffer (50 mM Tris, 150 mM NaCl, 1% Triton-X-100, 1% sodium deoxycholate, 0.1% SDS, 2 mM EDTA, 1X Roche Protease Inhibitors, PMSF). Lysate was then homogenized using a Cole-Parmer LABGEN 850 Homogenizer; 110 V at 13,000 rpm 3 × 20 s. Lysate was then sonicated and centrifuged at 4 °C at 15,000 g for 10 min. Protein concentration was measured using the BioRad DC Assay Kit. 50ug of protein extract was electrophoresed on TGX Stain-Free FastCast™ polyacrylamide gel (BioRad). Membrane was blocked in 0.05% TBST with 5% milk. Primary and secondary antibody details are provided in supplementary methods.

## Results

To investigate the structural pathology of the NMJ in slow- and fast-twitch motor units, soleus, gastrocnemius, and TA muscles were dissected from two mouse models of SBMA and evaluated for multiple features of NMJ pathology. The first mouse model, hereafter referred to as the *knock-in model* [74, 75], exhibits a progressive decrease in strength as early as 12 weeks of age, both neurogenic and myopathic muscle atrophy (with myopathic features preceding neurogenic atrophy), and fiber-type grouping [16, 58, 74, 75]. These mice also show partial fragmentation at the NMJ without denervation at 6 months of age [51]. The second mouse model used in this study, hereafter referred to as the *transgenic model*, exhibits slowly progressive motor deficits as early as 8 weeks of age and shows mutant androgen receptor (AR) intranuclear inclusions in spinal cord and skeletal muscle. It also exhibits muscle atrophy and shows no motor neuron loss in vivo, although extensive motor neuron death is observed in vitro upon hormone treatment [20, 45, 48, 50].

To understand the relationship between NMJ and muscle pathology, we aimed to evaluate both transgenic and knock-in mice at 6-months of age due to the presence of significant motor dysfunction and muscle atrophy in both models [5, 74, 75]. At 6-months of age, the knock-in mice show no motor neuron loss [34], and the transgenic mice show no motor neuron loss throughout their lifespan [5]. This cross-model comparison aimed to evaluate NMJ pathology at timepoints known to be devoid of motor neuron loss. However, due to the lack of NMJ pathology in the transgenic mice at 6-months of age (Additional File 1: Fig. S2), we evaluated the transgenic model at 1 year of age and the knock-in model at 6-months of age in order to better understand the extent of NMJ pathology in the absence of motor neuron loss.

### Gastrocnemius muscle shows significant neuromuscular junction pathology in two mouse models of SBMA.

**Fig. 1:**
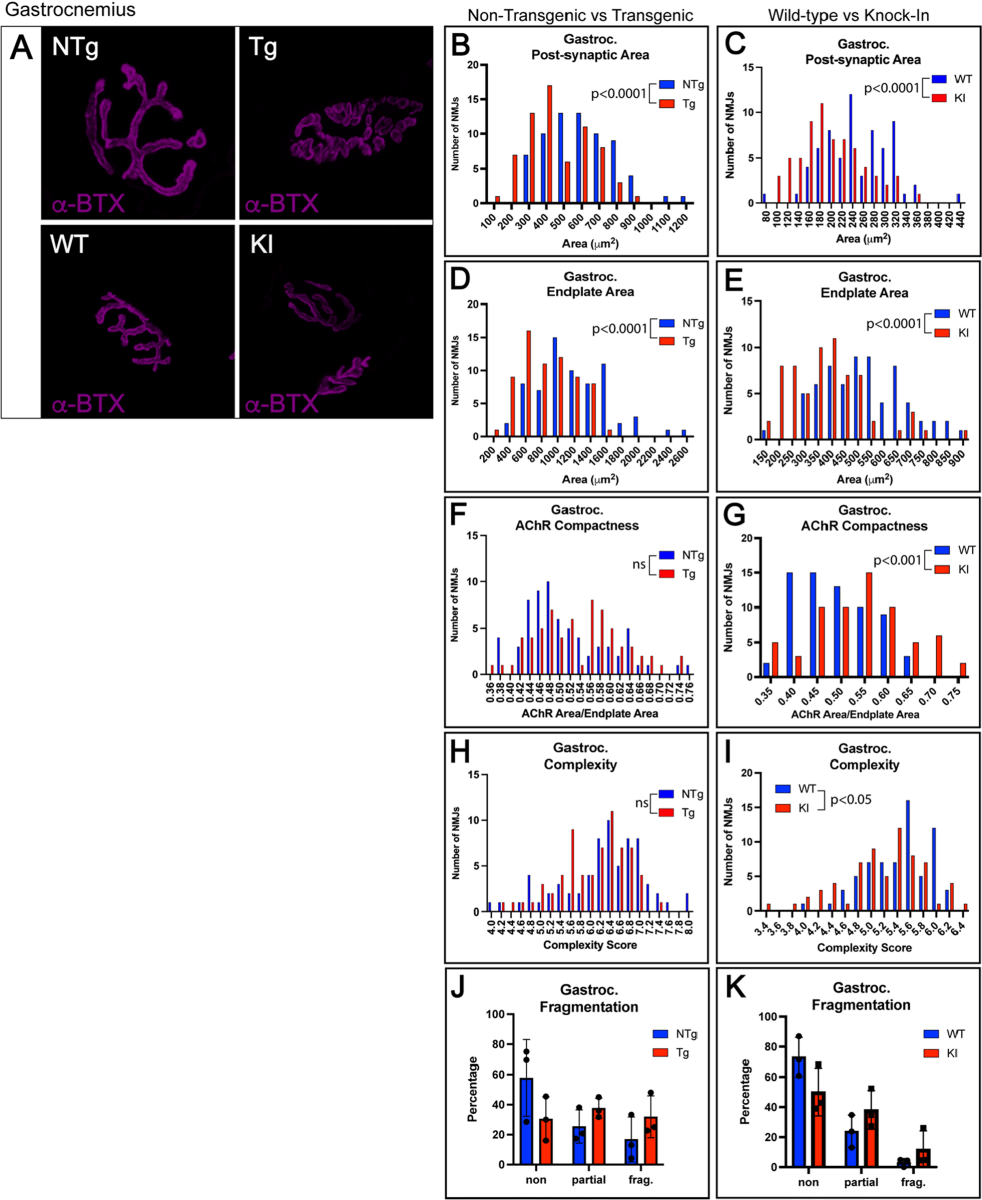
Gastrocnemius NMJs show significant alterations in post-synaptic area, endplate area, AChR compactness, and post-synaptic complexity in two mouse models of SBMA. **A** Post-synaptic membranes of NMJs from gastrocnemius muscle were labeled with fluorescently tagged α-bungarotoxin (α-BTX). Transgenic mice showed significantly decreased post-synaptic area (**B**; *p* < 0.0001) and endplate area (**D**; *p* < 0.0001), and a trending reduction in post-synaptic complexity (**H**). AChR compactness showed a trending increase in transgenic mice compared to NTg controls (**F**). While the percentage of fragmented endplates was slightly increased, this did not reach statistical significance (**J**). Knock-in mice showed significant decreases in post-synaptic area (**C**; *p* < 0.0001), endplate area (E; p < 0.0001), and post-synaptic complexity (**I**; *p* < 0.05). AChR compactness was significantly increased in knock-in mice compared to wild-type littermates (**G**; *p* < 0.001). While the percentage of fragmented endplates in knock-in mice was increased, this did not reach statistical significance (**K**). Mann–Whitney Test was used to evaluate statistical significance for distributions. Student’s t-test was used to evaluate fragmentation. *NTg*, Non-transgenic; *Tg* Transgenic; *WT* Wild-type; *KI* Knock-in

**Fig. 2:**
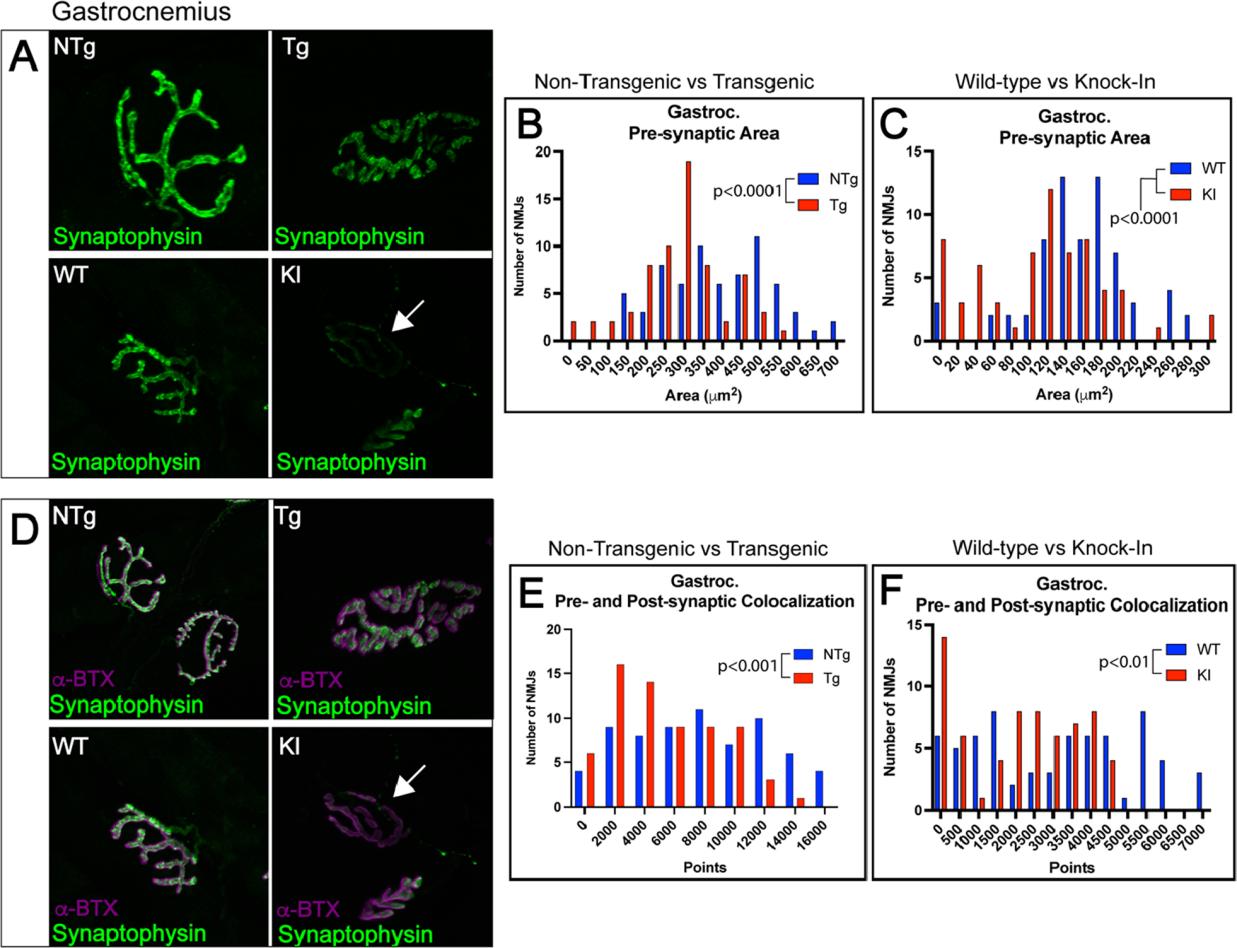
Gastrocnemius NMJs show significant decreases in pre-synaptic area and pre- and post-synaptic colocalization in transgenic and knock-in models of SBMA. Pre-synaptic terminals were stained with synaptic vesicle marker synaptophysin and measured for pre-synaptic area and colocalization with post-synaptic membrane, labeled with fluorescently tagged a-bungarotoxin. Arrows point to NMJ with low synaptophysin staining. Synaptophysin image in A were merged with α-BTX images for panel D. **A** Gastrocnemius NMJs showed significantly decreased pre-synaptic area in transgenic and knock-in mice (**B** NTg vs Tg *p* < 0.0001; **C** WT vs KI *p* < 0.0001). **D** Gastrocnemius NMJs showed significantly decreased pre- and post-synaptic colocalization in transgenic and knock-in mice (E NTg vs Tg, *p* < 0.001; F WT vs KI, *p* < 0.01). Mann–Whitney Test was used to evaluate statistical significance for distributions. Abbreviations: NTg, non-transgenic; Tg, transgenic; WT, wild-type; KI, knock-in

To understand how mutant AR expression affects the NMJ, the primarily fast-twitch gastrocnemius muscles from both mouse models were evaluated for NMJ pathology (Additional File 1: Fig. S1). Post-synaptic area was found to be substantially decreased in both transgenic and knock-in mice compared to control littermates (Fig. 1A–C). Endplate area was also substantially decreased in both models (Fig. 1D, E). AChR compactness, calculated by dividing post-synaptic area by endplate area, was increased in both models but only reached statistical significance in the knock-in model (Fig. 1F, G). Both models showed a decrease in endplate complexity, but this only reached statistical significance in knock-in NMJs (Fig. 1H, I). Both models showed a modest increase in the percentage of fragmented NMJs; however, this did not reach statistical significance (Fig. 1J, K), highlighting the value of exploring quantitative measurements of NMJ pathology. Presynaptic area, measured using localization of synaptic vesicle protein synaptophysin, was significantly reduced in both models (Fig. 2A–C). Pre- and post-synaptic colocalization was also decreased in both models (Fig. 2D–F). These detailed quantitative data (Additional File 1: Table S1) provide evidence for significant pre- and post-synaptic pathology at the NMJs of the primarily fast-twitch gastrocnemius muscle in two mouse models of SBMA.

**Fig. 3:**
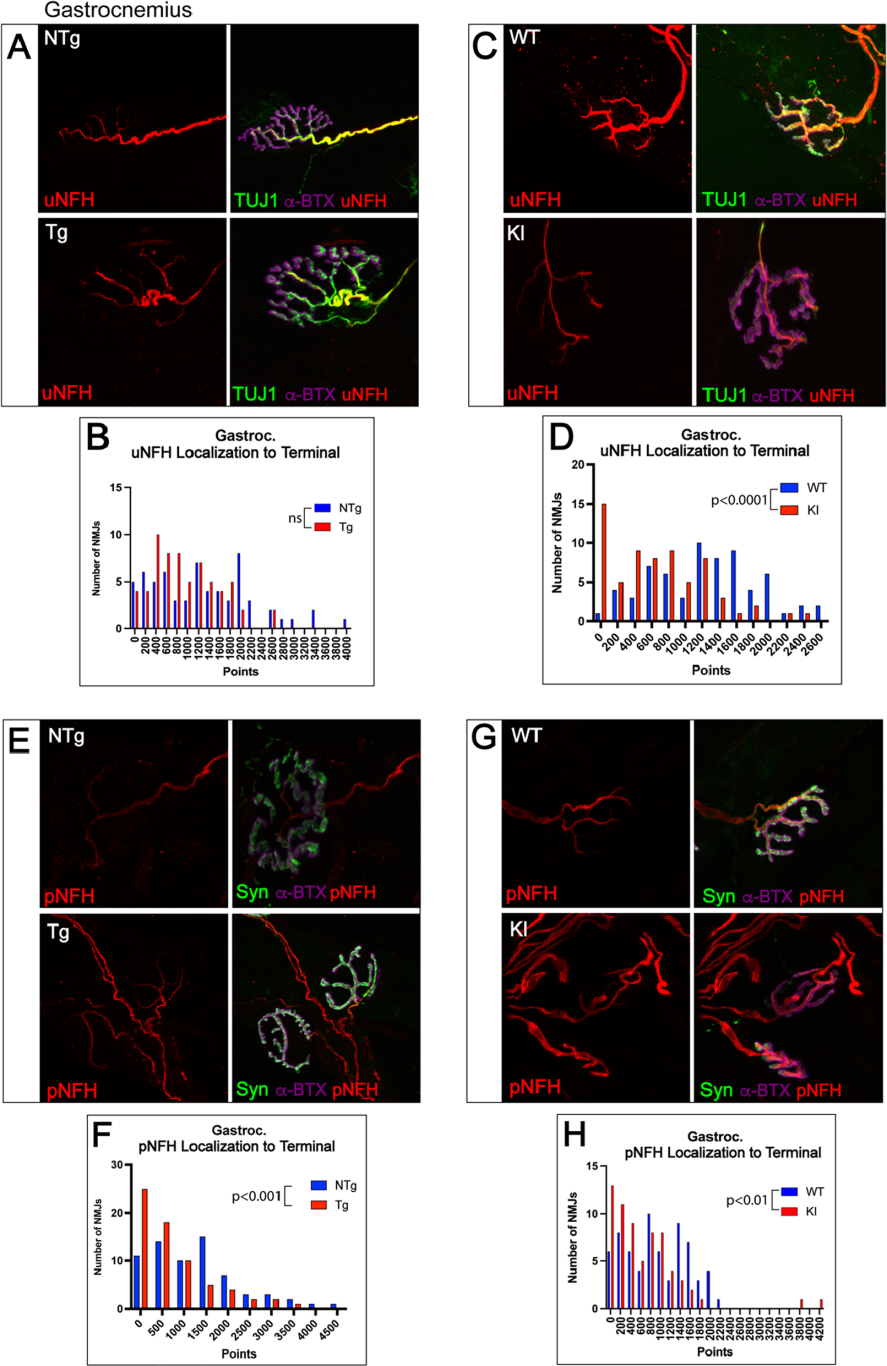
Gastrocnemius NMJs show significant changes in localization of phosphorylated and unphosphorylated neurofilament heavy chain to the axon terminal in two mouse models of SBMA. (**A**, **C**, **E**, **G**) To evaluate localization of cytoskeletal structural element NFH to the axon terminal, NMJs from gastrocnemius were stained with α-BTX, synaptophysin, and SMI31 (phospho-NFH) or α-BTX, TUJ1 (BIII-tubulin), and SMI32 (unphospho-NFH) and evaluated for colocalization with synaptophysin or α-BTX, respectively. **B**, **D** Both transgenic (trending, *p* = 0.0618) and knock-in mice (*p* < 0.0001) showed decreased uNFH localization to the terminal. **F**, **H** Both transgenic (*p* < 0.001) and knock-in (*p* < 0.01) mice also showed significantly decreased pNFH colocalization with the terminal. Mann–Whitney Test was used to evaluate statistical significance. *uNFH* Unphosphorylated neurofilament heavy chain; *pNFH* Phosphorylated neurofilament heavy chain; *NTg* Non-transgenic; *Tg* Transgenic; *WT* Wild-type; *KI* Knock-in

Previous studies in a transgenic model of SBMA revealed a significant reduction in unphosphorylated neurofilament heavy chain (uNFH) in the cell bodies of spinal motor neurons [5]. At the axon terminal of NMJs in the gastrocnemius, uNFH intensity was significantly decreased in both models (Additional File 1: Fig. S3). Moreover, localization of uNFH to the axon terminal, as measured by colocalization with fluorescently tagged α-BTX, was decreased in gastrocnemius of the knock-in model (Fig. 3C, D), and trended lower in the transgenic model (Fig. 3A, B). Since NFH is increasingly phosphorylated as it travels in the anterograde direction towards the axon terminal, we also evaluated phosphorylated NFH (pNFH) localization to the axon terminal through colocalization with synaptophysin (Fig. 3E, G). In both mouse models, although pNFH intensity was unchanged (Additional File 1: Fig. S3), pNFH colocalization with the pre-synaptic terminal was decreased (Fig. 3F). These data provide evidence for a significant deficit in NFH at the axon terminal, which correlates with the substantial pre- and post-synaptic alterations in NMJs of the gastrocnemius.

### Fast-twitch tibialis anterior motor units show significant changes in the post-synaptic membrane of the NMJ.

**Fig. 4:**
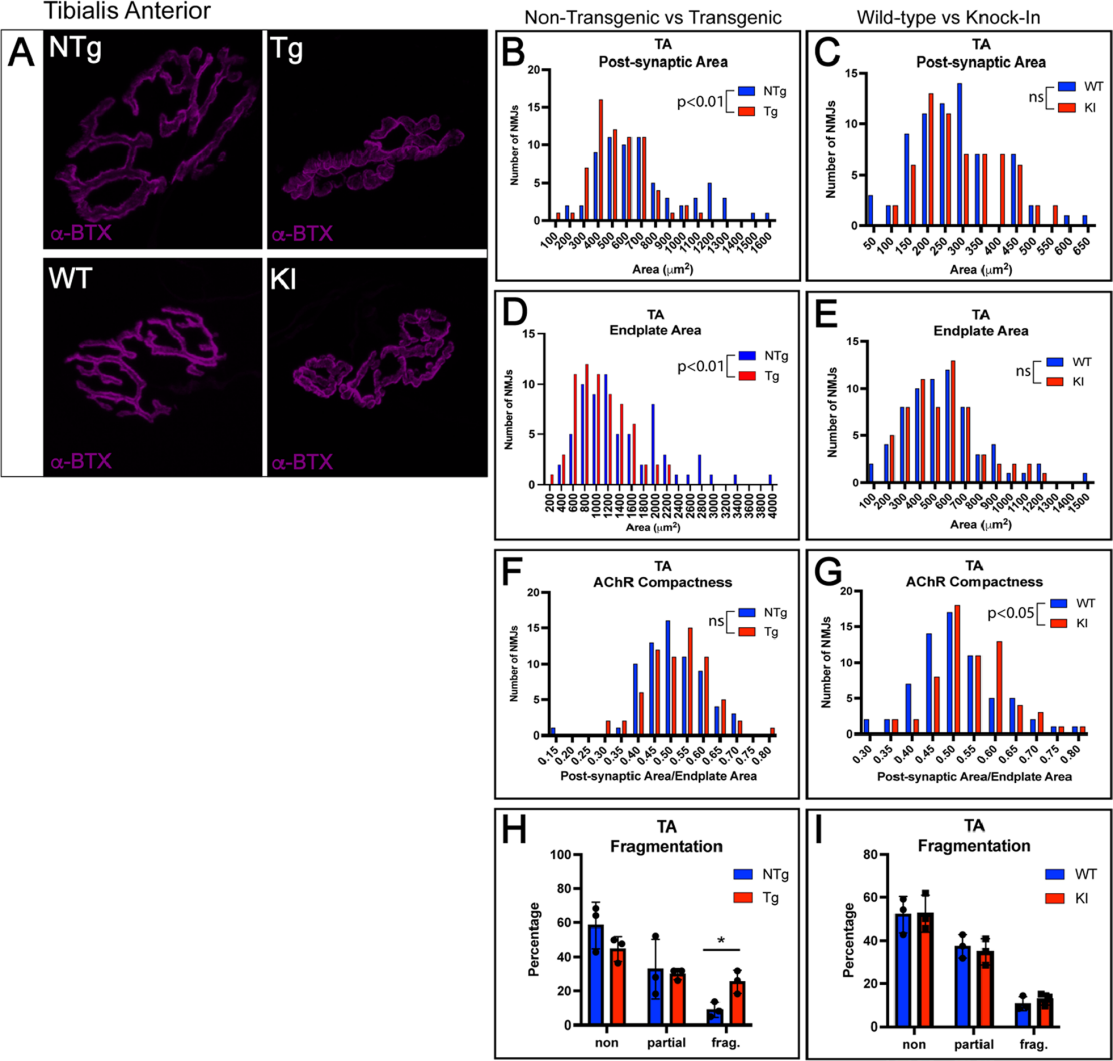
Fast-twitch tibialis anterior motor units show significant changes in post-synaptic membrane of NMJ. Post-synaptic membranes of NMJs from tibialis anterior (TA) muscle were labeled with fluorescently tagged α-bungarotoxin (α-BTX). **A** Transgenic mice showed significantly decreased post-synaptic area (**B**; *p* < 0.01) and endplate area (**D**; *p* < 0.01) without an increase in AChR compactness (**F**). Transgenic mice showed a significant increase in the percentage of fragmented NMJs (**H**; *p* < 0.05). Knock-in mice did not show an increase in fragmentation (I), post-synaptic area (C) or endplate area (E) but did show an increase in AChR compactness (G; p < 0.05). Mann–Whitney Test was used to evaluate statistical significance for distributions. Student’s t-test was used to evaluate fragmentation. Abbreviations: *Ntg*, non-transgenic; *Tg*, transgenic; *WT*, wild-type; *KI*, knock-in

To further evaluate the pathology of fast-twitch motor units in these two mouse models of SBMA, we evaluated NMJs in fast-twitch TA using the same procedure described for the analysis of gastrocnemius. In the transgenic mouse model, TA NMJs showed a significant decrease in post-synaptic area and endplate area, without a corresponding increase in acetylcholine receptor compactness (Fig. 4A, B, D, F). However, unlike gastrocnemius NMJs, TA NMJs in the transgenic model showed a significant increase in fragmentation, a well-studied indicator of NMJs that are degenerating or remodeling [63]. In comparison, NMJs of knock-in TA showed a significant change only in AChR compactness (Fig. 4G), despite showing no significant differences in post-synaptic area or endplate area (Fig. 4C, E). Knock-in TA NMJs showed no change in fragmentation (Fig. 4I). There were no significant changes in endplate complexity or in any pre-synaptic markers, including area and pre- and postsynaptic colocalization, in the TA of either model (Additional File 1: Fig. S4). These data (Additional File 1: Table S1) provide evidence for more limited NMJ pathology in the NMJs of TA, observed primarily in the post-synaptic membrane, in contrast to the more substantial pre- and post-synaptic pathology seen in the gastrocnemius muscle, despite the fact that both muscles contain primarily fast-twitch muscle fibers.

**Fig. 5:**
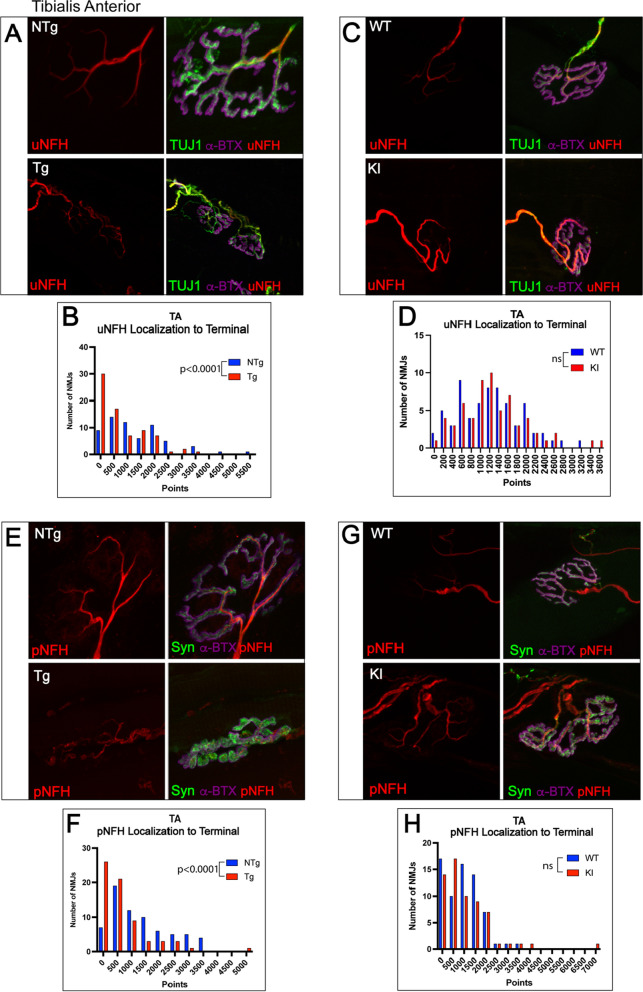
Neurofilament heavy chain (NFH) localization to the axon terminal is altered in transgenic but not knock-in mice in tibialis anterior. **A**, **C**, **E**, **G** To evaluate localization of cytoskeletal structural element NFH to the axon terminal, NMJs from tibialis anterior were stained with α-BTX, synaptophysin, and SMI31 (phospho-NFH) or α -BTX, TUJ1 (BIII-tubulin, and SMI32 (unphospho-NFH) and evaluated for colocalization with the synaptophysin or α-BTX, respectively. Consistent with the trends seen in pre- and post-synaptic NMJ measurements, transgenic mice showed significantly decreased uNFH localization to the axon terminal (**B**; *p* < 0.0001) and pNFH colocalization with terminal (**F**; *p* < 0.0001) while knock-in mice did not show changes in either of these measurements (**D**, **H**). Mann–Whitney Test was used to evaluate statistical significance. *uNFH* Unphosphorylated neurofilament heavy chain; *pNFH* Phosphorylated neurofilament heavy chain; *NTg* Non-transgenic; *Tg* Transgenic; *WT* Wild-type; *KI* Knock-in

Although pathology at the NMJ of TA muscles in transgenic mice was observed primarily at the post-synaptic membrane (post-synaptic area, endplate area, and fragmentation), alterations in pNFH intensity (Additional File 1: Fig. S5) and localization to the axon terminal (Fig. 5E, F) were present at the pre-synaptic membrane of NMJs of the TA in transgenic mice. In addition, uNFH localization to the pre-synaptic terminal was also significantly decreased in this model (Fig. 5A, B), providing evidence for general NFH depletion at the NMJ. In contrast, the NMJs of TA in knock-in mice showed a significant *increase* in pNFH staining intensity compared to littermate controls (Additional File 1: Fig. S5) and did not show changes in uNFH intensity or localization of pNFH or uNFH to the pre-synaptic terminal (Fig. 5C, D, G, H). The changes in NFH at TA NMJs appear to correlate with the severity of NMJ pathology observed in both models – the transgenic model, which showed multiple signs of NMJ structural deficits, also showed substantial decreases in NFH, while the knock-in model, which only showed alterations in AChR compactness, showed increased pNFH intensity and no changes in colocalization of NFH with the pre-synaptic terminal.

### Slow-twitch soleus shows less NMJ pathology than fast-twitch muscles

**Fig. 6:**
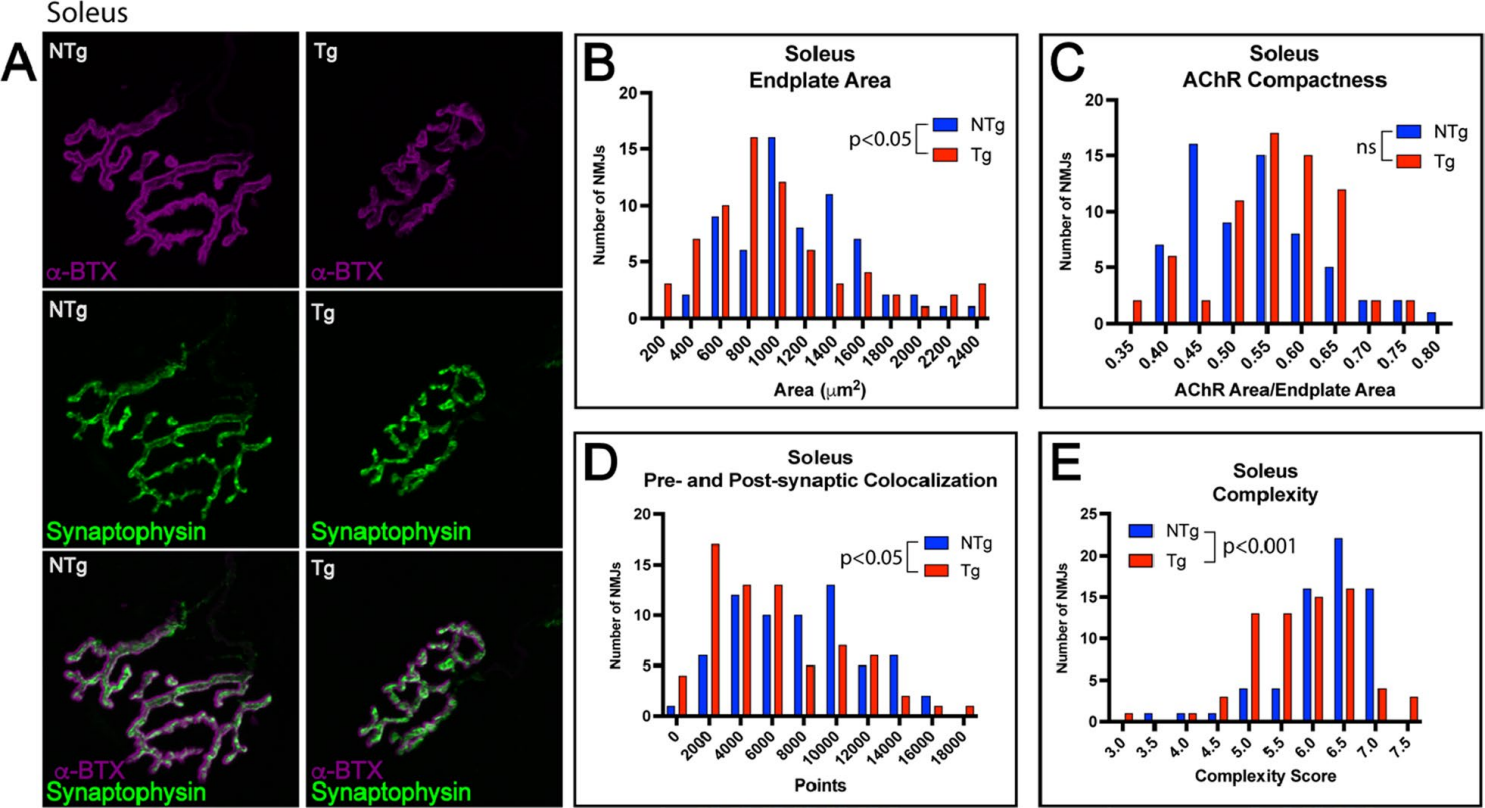
Slow-twitch soleus shows moderate NMJ pathology in transgenic, but not in knock-in, model of SBMA. Pre-synaptic membranes of NMJs from soleus muscle were stained with synaptic vesicle marker synaptophysin and post-synaptic membranes were labeled with fluorescently tagged α-bungarotoxin (α-BTX; **A**). Transgenic mice showed a significant decrease in endplate area (**B**; *p* < 0.05), pre- and post-synaptic colocalization (**D**; *p* < 0.05), and post-synaptic complexity (**E**; *p* < 0.001). AChR compactness trended to an increase in transgenic mice, but this did not reach statistical significance (**C**). Knock-in mouse NMJs of the soleus did not show any significant changes in NMJ pathology compared to WT littermates (Additional File 1: Fig. S6). Mann–Whitney Test was used to evaluate statistical significance. *NTg* Non-transgenic; *Tg* Transgenic; *WT* Wild-type; *KI* Knock-in

**Fig. 7:**
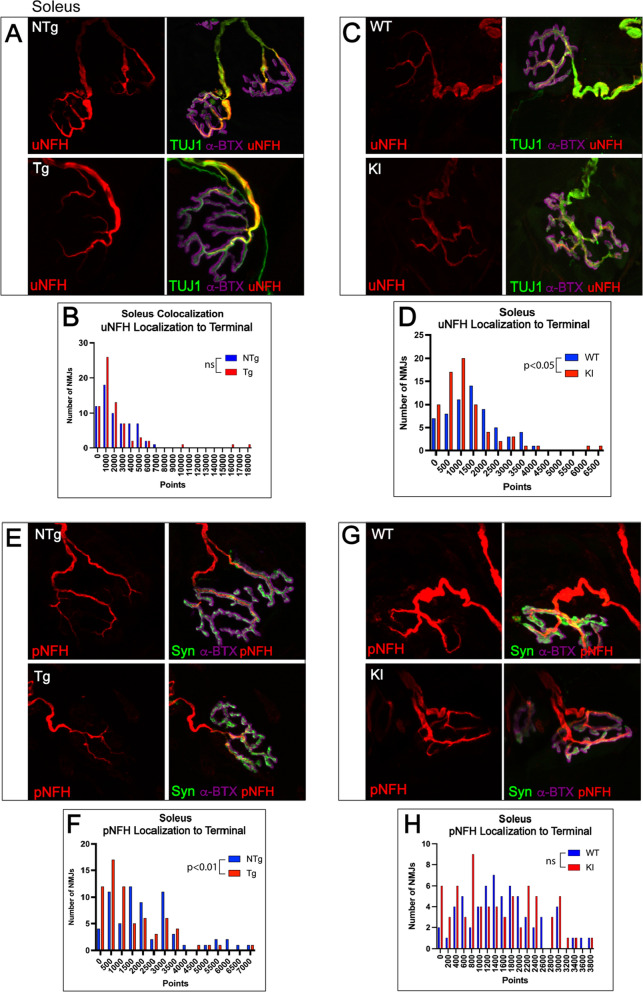
Soleus NMJs show significant decrease in localization of NFH to the axon terminal. **A**, **C**, **E**, **G** To evaluate localization of cytoskeletal structural element NFH to the axon terminal, NMJs from tibialis anterior were stained with α-BTX, synaptophysin, and SMI31 (phospho-NFH) or α-BTX, TUJ1 (BIII-tubulin, and SMI32 (unphospho-NFH) and evaluated for colocalization with the synaptophysin or α-BTX, respectively. **B** Transgenic mice showed no changes in uNFH localization to the terminal; however, knock-in mice showed a significant decrease in uNFH localization with the terminal (**D**; *p* < 0.05). **D** In contrast, pNFH colocalization with the terminal was significantly decreased in transgenic mice (**E**; *p* < 0.01) but not in knock-in mice (**F**). Mann–Whitney Test was used to evaluate statistical significance. *uNFH* Unphosphorylated neurofilament heavy chain; *pNFH* Phosphorylated neurofilament heavy chain; *NTg* Non-transgenic; *Tg* Transgenic; *WT* Wild-type; *KI* Knock-in

To evaluate differences between fast-twitch and slow-twitch motor unit pathology, we evaluated NMJs of the slow-twitch soleus muscle in both mouse models of SBMA. In transgenic mice, soleus NMJs exhibited modestly decreased endplate area, pre- and postsynaptic colocalization, and endplate complexity (Fig. 6), with a trend toward an increase in AChR compactness. There were no significant changes in postsynaptic area, presynaptic area, or fragmentation (Additional File 1: Fig S6). Transgenic mice also showed a significant decrease in pNFH colocalization with the pre-synaptic terminal, without alterations in pNFH intensity, uNFH intensity, or uNFH localization to the terminal (Fig. 7A, B, E, F; Additional File 1: Fig. S7). Moreover, soleus NMJs from knock-in mice exhibited no significant changes in any of the evaluated parameters (Additional File 1: Fig. S6), except a decrease in uNFH intensity and uNFH localization to the pre-synaptic terminal (Fig. 7C, D, G, H; Additional File 1: Fig. S7). These data support the hypothesis that slow-twitch motor units exhibit substantially less NMJ pathology than fast-twitch motor units, as both models exhibited stark differences in NMJ pathology between fast- and slow-twitch motor units (Additional File 1: Table S1).

### AR expression is not significantly different in muscles of transgenic and knock-in mice.

To further understand possible mechanisms driving the heightened NMJ pathology in transgenic mice compared to knock-in mice in both TA and soleus muscles, we considered the differences in AR expression between the two models. AR expression is substantially greater in the spinal cord of transgenic mice compared to non-transgenic mice [5, 77]. Because knock-in mice express polyglutamine-expanded AR under mouse regulatory gene elements, AR expression in these mice is equal to that in non-transgenic mice which endogenously express non-expanded AR. Additionally, while the transgenic mice primarily express polyglutamine-expanded AR in the central nervous system, polyglutamine-containing intranuclear inclusions are seen in muscle (Additional File 1: Fig. S8). We evaluated AR protein expression in gastrocnemius muscle fibers from transgenic and knock-in mice (Additional File 1: Fig. S8) and observed no significant differences between the two models in AR intensity. Therefore, the differences in NMJ pathology between transgenic and knock-in mice are unlikely to be attributed to elevated AR expression in transgenic muscle. Our results thus suggest that the increased levels of polyglutamine-expanded AR in the spinal cord of the transgenic mice likely contribute to the greater NMJ pathology in transgenic soleus and TA muscles.

### Acetylcholine receptor subunit expression is altered in SBMA muscle

Previous studies revealed increased AChR gamma mRNA in transgenic and knock-in mouse models of SBMA, corresponding to electrophysiological changes in NMJ transduction in extensor digitorum longus and levator ani muscles [71]; therefore, we explored whether AChR gamma protein levels correlated with NMJ pathology. Nicotinic AChRs are composed of five subunits – two alpha, one beta, one delta, and either one gamma or epsilon subunit, depending on the developmental stage of the organism, with the epsilon subunit expressed at mature NMJs and the gamma subunit expressed in fetal or denervated NMJs [44, 69]. Gamma subunits are also expressed at the NMJs of intrafusal muscle fibers [15]. We evaluated AChR gamma protein expression by Western blot and observed a trend toward decreased expression in the soleus of both transgenic and knock-in mice (Additional File 1: Fig. S9A). In contrast to these observations in the soleus, the TA exhibited increased AChR gamma levels in knock-in mice, which similarly trended higher in transgenic mice (Additional File 1: Fig. 9B). Gastrocnemius, however, did not show any alterations in AChR gamma protein levels, despite exhibiting even greater NMJ pathology than TA (Additional File 1: Fig. S9C). Thus, in the absence of a correlation between AChR gamma expression and NMJ pathology, it is unlikely that heightened AChR gamma expression plays a role in or serves as a marker of motor unit vulnerability in these models.

### Muscle fibers in gastrocnemius, tibialis anterior, and soleus muscles exhibit a shift to increased oxidative metabolism and decreased cross-sectional area

**Fig. 8:**
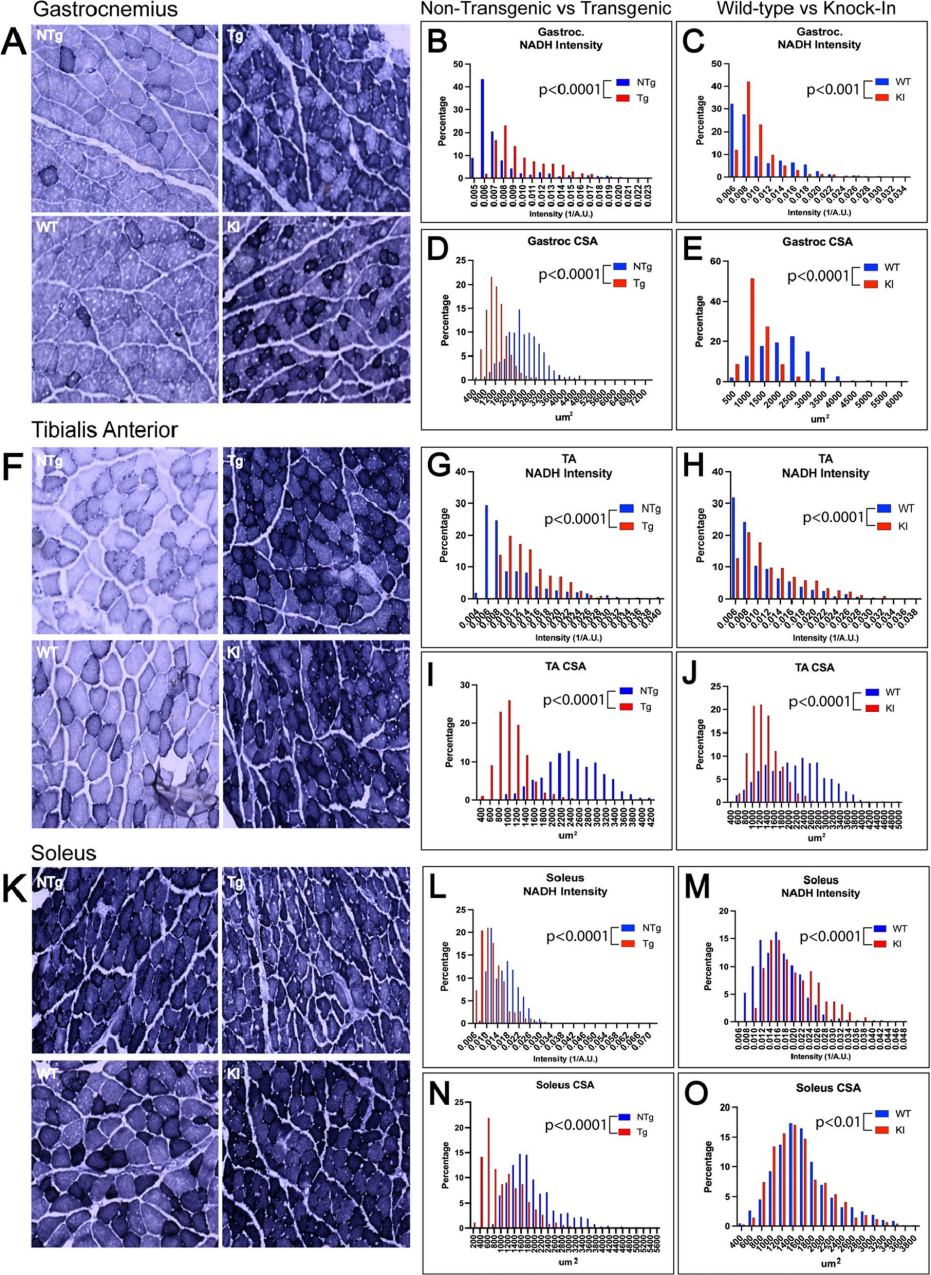
Muscle fibers in gastrocnemius, tibialis anterior, and soleus muscles show an increase in NADH-diaphorase staining and a decrease in cross-sectional area. Muscle sections were stained with nicotinamide adenine dinucleotide (NADH)-disaphorase to assess oxidative state. **A**–**C** Gastrocnemius showed an increase in NADH staining intensity in both transgenic (*p* < 0.0001) and knock-in (*p* < 0.001) mice. **D**, **E** This was accompanied by a decrease in cross sectional area (CSA) in both models (transgenic *p* < 0.0001; knock-in *p* < 0.0001). **F** Tibialis anterior also showed a significant increase in NADH staining intensity in both models (**G**, **H**; transgenic *p* < 0.0001; knock-in *p* < 0.0001) along with a significant reduction in cross-sectional area (**I**, **J**; transgenic *p* < 0.0001; knock-in *p* < 0.0001). **K** In soleus, NADH intensity was shifted in opposite directions across the two models. **L**, **M** The transgenic model showed a significant decrease in NADH staining intensity (**L**; *p* < 0.0001) while knock-in mice showed an increase in staining intensity (**M**, *p* < 0.0001). (N, O) Both models showed a decrease in cross sectional area, although this is more severe in transgenic mice (transgenic *p* < 0.0001; knock-in *p* < 0.01). Mann–Whitney Test was used to evaluate statistical significance. *NTg* Non-transgenic; *Tg* Transgenic; *WT* Wild-type; *KI* Knock-in; *CSA*, Cross-sectional area

Because the NMJ is the chemical synapse between the motor neuron and the muscle fibers it innervates, it is important to consider muscle fiber changes that occur concomitant with NMJ pathology. Using nicotinamide adenine dinucleotide (NADH)-diaphorase staining, we evaluated cross-sections of gastrocnemius for evidence of muscle atrophy and fiber-type switching (Fig. 8A). As expected, due to the high proportion of fast-twitch glycolytic fibers in gastrocnemius muscles, non-transgenic/wild-type gastrocnemius muscle fibers showed light staining with little pigment deposition. Both transgenic (Fig. 8A, B) and knock-in (Fig. 8A, C) gastrocnemius muscle fibers showed substantially increased staining intensity, suggesting a shift to more oxidative metabolism. Moreover, the cross-sectional area of gastrocnemius muscle fibers was significantly decreased in both transgenic (Fig. 8A, D) and knock-in mice (Fig. 8A, E). These data reveal significant alterations in muscle fiber composition along with evidence of muscle atrophy in gastrocnemius of both SBMA mouse models.

To determine if the more modest NMJ pathology in TA compared to gastrocnemius, described above (Figs. 3, 4, Additional File 1: Figs. S3 and S4), could be due to underlying differences in muscle pathology and to further evaluate fast-twitch motor unit vulnerability in the knock-in and transgenic mouse models, we also performed NADH-diaphorase staining of TA muscles (Fig. 8F). Both models showed significantly increased NADH staining intensity in TA, providing evidence for a similar oxidative shift in TA muscle fibers (Fig. 8F–H). This was accompanied by a significant decrease in cross-sectional area (Fig. 8F, I, J), as observed in gastrocnemius muscle. In both TA and gastrocnemius muscles, the observed shift to oxidative metabolism was more substantial in transgenic mice than in knock-in mice (Additional File 1: Table S2).

To further understand differences in fast- and slow-twitch motor unit pathology in SBMA, we also evaluated histological changes in soleus muscle (Fig. 8K). In knock-in mice, soleus muscle fibers showed darker staining, representing further increased oxidative metabolism (Fig. 8K, M), as seen in gastrocnemius and TA muscles in both models. However, unlike knock-in mice, transgenic mice showed a significant decrease in NADH staining (Fig. 8K, L). This unexpected result could result from an oxidative-to-glycolytic shift as a result of altered metabolism [49] or from reduced oxidative capacity due to disease progression. It is worth noting that, while soleus muscles of both the knock-in and transgenic mouse models showed significant decreases in cross-sectional area, a much more substantial decrease was observed in transgenic mice (Fig. 8K, N) compared to knock-in mice (Fig. 8K, O; Additional File 1: Table S2).

The combined histological and structural analyses of muscle fibers in the two mouse SBMA mouse models used here indicate that muscles composed of primarily fast-twitch fibers exhibit a substantial shift from glycolytic to oxidative metabolism, with a corresponding decrease in cross-sectional area. Slow-twitch soleus muscles, in contrast, exhibited an oxidative shift that was observed only in the knock-in model. Irrespective of the oxidative state of the individual muscle fibers, all muscle fibers exhibited substantially decreased cross sectional area, which was more severe in the transgenic mice. The greater muscle pathology observed in the transgenic model correlates with the increased NMJ pathology in this model, possibly as a result of the older age of these mice.

### Muscle cytoskeletal protein expression is significantly altered in transgenic and knock-in mice

**Fig. 9:**
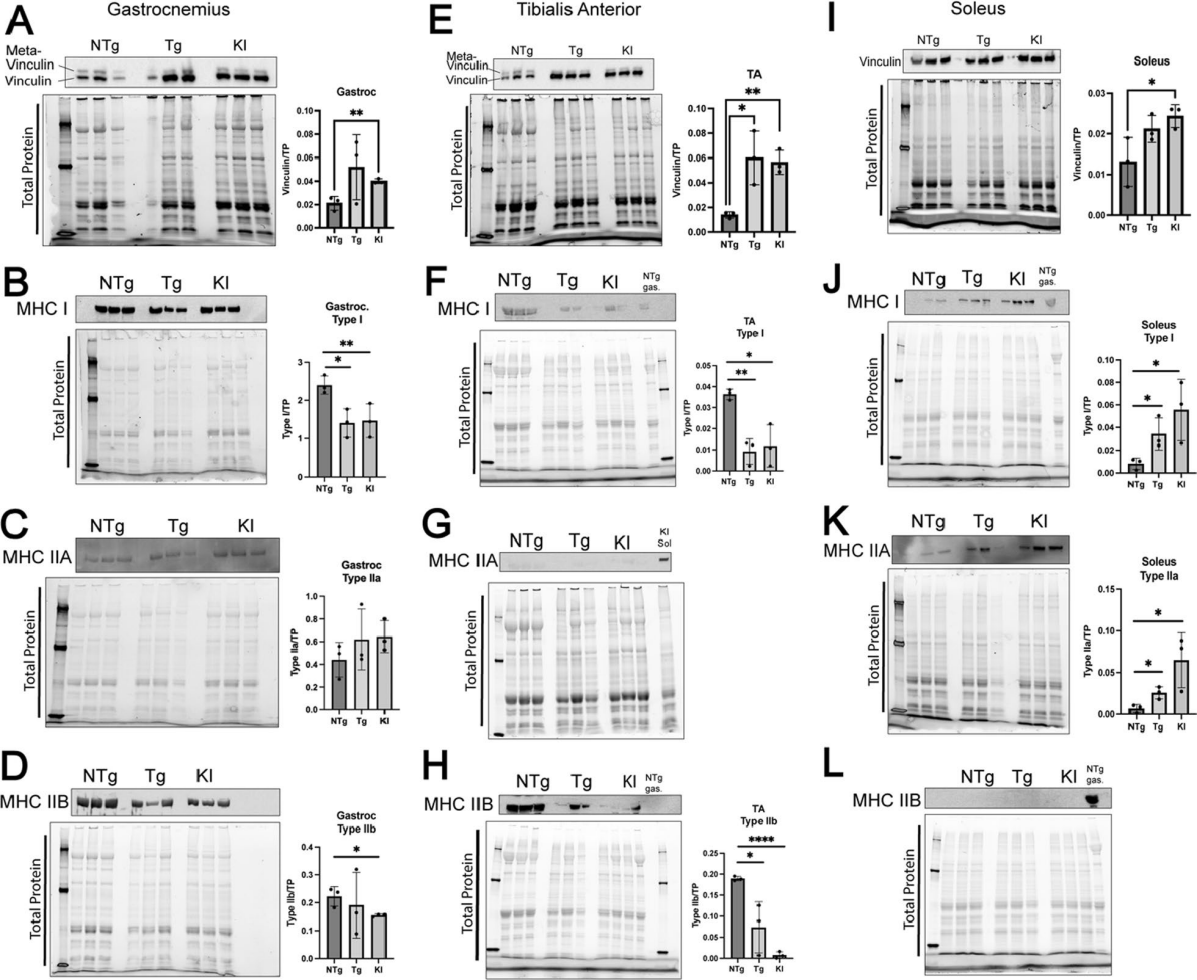
Cytoskeletal elements vinculin and myosin heavy chain (I, IIA, IIB) are altered in fast- and slow-twitch muscles. Cytoskeletal protein vinculin was increased in **A** gastrocnemius, **E** tibialis anterior, and **I** soleus muscles in 12-month-old transgenic and 9-month-old knock-in mouse models of SBMA. In tibialis anterior, both transgenic (*p* < 0.05) and knock-in mice (*p* < 0.01) showed significant increases in vinculin. Vinculin increase only reached statistical significance in knock-in mice in soleus (*p* < 0.05) and gastrocnemius (*p* < 0.01). **B** Gastrocnemius muscle showed a significant decrease in MHC I in both transgenic (*p* < 0.05) and knock-in mice (*p* < 0.05). **C** MHC IIA was unaltered in gastrocnemius of transgenic and knock-in mice. **D** Knock-in mice showed a significant decrease in MHC IIB levels (*p* < 0.05) in gastrocnemius, which did not occur in transgenic mice. **F** Tibialis anterior showed a significant decrease in MHC I in both transgenic (*p* < 0.01) and knock-in (*p* < 0.05) mice. **G** Tibialis anterior showed undetectable levels of MHCIIA. Knock-in soleus lysate used as positive control. **H** Tibialis anterior showed a significant decrease in MHC IIB in both transgenic (*p* < 0.01) and knock-in (*p* < 0.0001) mice. **J** Soleus muscle showed a significant increase in MHC I in both transgenic (*p* < 0.05) and knock-in mice (*p* < 0.05). **K** Soleus muscle also showed an increase in MHC IIA in both transgenic (*p* < 0.05) and knock-in (*p* < 0.05) mice. **L** As expected, soleus did not express MHC IIB in non-transgenic, transgenic, or knock-in mice. NTg gastrocnemius lysate used as positive control. Student’s t-test was used to evaluate statistical significance. *NTg* Non-transgenic; *Tg* Transgenic; *WT* Wild-type; *KI*, Knock-in; *MHC I* Myosin heavy chain type 1; *MHC II* Myosin heavy chain type 2A; *MHC IIB* Myosin heavy chain type 2B; *p < 0.05; **p < 0.01, ****p < 0.0001

Vinculin, a cytoskeletal component of striated muscle, is present in significantly greater quantities in slow-twitch, oxidative, muscle fibers than in fast-twitch, glycolytic, muscle fibers, and meta-vinculin, a vinculin splice isoform, is present only in fast-twitch muscle fibers of wild-type mice (Fig. 9) [8]. Denervation of TA in multiple mouse models resulted in increased vinculin levels [57], leading us to question whether vinculin levels in the three muscles evaluated here were altered. Western blot analysis of gastrocnemius, TA, and soleus revealed increased vinculin levels in all three muscles of knock-in mice and in TA of transgenic mice (Fig. 9A, E, I), with a trending increase in gastrocnemius and soleus of the transgenic mice. This increase in vinculin expression is consistent with a shift in these muscles to a more oxidative metabolism, as vinculin is highly expressed in slow-twitch muscle fibers to enable sustained contraction [8].

To further understand a possible fast-to-slow fiber-type switch and the glycolytic-to-oxidative metabolic shift, we evaluated myosin heavy chain (MHC) isoform expression in soleus, TA, and gastrocnemius of both mouse models. Contrary to our hypothesis that gastrocnemius and TA muscles would exhibit a switch to increased expression of an oxidative, slow-type muscle MHC (MHC type I), gastrocnemius and TA showed a significant decrease in MHC I expression in both transgenic and knock-in muscle (Fig. 9B, F). As expected, both of these primarily fast-twitch muscles showed significantly decreased fast-twitch MHC isoform, MHC IIB, although in gastrocnemius this only reached significance in the knock-in mice (Fig. 9D, H). MHC IIA, primarily found in oxidative fast-twitch fibers, was not significantly changed in gastrocnemius (Fig. 9C) and was undetectable in TA. Together, these data point to a decrease in overall MHC protein expression rather than a switch in contractile fiber-type. Soleus muscles in both transgenic and knock-in mice showed a significant increase in MHC I and MHC IIA expression compared to non-transgenic controls (Fig. 9J, K). As expected, the soleus of all three genotypes did not express fast-twitch myosin heavy chain, MHC IIB.

## Discussion

Spinal and bulbar muscular atrophy (SBMA) is a debilitating neuromuscular neurodegenerative disease for which there is no cure [13]. Symptoms reflect neuromuscular system degeneration and implicate dysfunctional communication at the primary functional synapse of the motor unit, the neuromuscular junction (NMJ). It has been shown previously in both SBMA and ALS that fast-twitch motor units are more susceptible to degeneration than slow-twitch motor units [19, 42, 73]. However, it is unknown whether pathology at the NMJ differs between motor unit subtypes in SBMA. Based on previous data showing a loss of fast-twitch muscle fibers in SBMA patients and SBMA mouse models [7, 23, 58, 73], we hypothesized that TA would show significant NMJ pathology due to its classification as a fast-twitch muscle, and that fast-twitch gastrocnemius would show equal or less pathology than TA due to the higher percentage of type I (slow) and IIa (fast-oxidative) fibers [3]. Correspondingly, we hypothesized that slow-twitch soleus muscle would show little to no evidence of NMJ pathology.

In this study, our data revealed, as expected, that fast-twitch motor units of the gastrocnemius and TA showed more severe NMJ pathology than slow-twitch motor units of the soleus, and that this difference correlated with metabolic and structural changes in the innervated muscle fibers. However, our observations suggest a more complex interpretation than originally hypothesized, as NMJ pathology in the TA was limited to post-synaptic changes only in the transgenic model and was less severe than NMJ pathology in the gastrocnemius, even though both are considered fast-twitch muscles. One possible explanation for these observations is the developmental difference between gastrocnemius and TA in AChR clustering. It is known that, in response to disease and/or injury, both myofibers and motor neurons reinitiate the developmental program [21, 22, 78]. During development, TA myofibers exhibit fast-synapsing (FaSyn) characteristics, while gastrocnemius and soleus exhibit delayed-synapsing (DeSyn) features [53]. FaSyn TA myofibers focally cluster AChRs and align motor nerves with AChR clusters at a substantially faster rate during development than DeSyn gastrocnemius and soleus myofibers, and differences in AChR clustering occur even in the absence of an innervating motor neuron [53]. This developmental difference impacted disease severity in a model of MuSK-induced myasthenia gravis, in which NMJs of DeSyn muscles showed significantly decreased post-synaptic area compared to NMJs of FaSyn muscles [70]. The ability of TA (FaSyn) to focally cluster AChRs at a higher rate could have an impact on its ability to respond to disease pathology when the developmental program is re-initiated, resulting in less NMJ pathology than its fast-twitch counterpart, gastrocnemius (DeSyn). Of note, TA was the only muscle evaluated that showed upregulated AChR gamma (Additional File 1: Fig. S9), which is known to be required for AChR clustering during development [37]. We hypothesize that the upregulated expression of AChR gamma in diseased TA is related to its classification as a FaSyn muscle. Validation of this idea that intersecting developmental and degenerative mechanisms contribute to NMJ pathology in SBMA requires further validation.

NMJs in all three muscles evaluated in this study showed significant changes in NFH levels and/or distribution. NFH is one of four proteins that make up neurofilament, a cytoskeletal protein necessary for structural integrity and axon diameter [40]. While it is known that phospho-NFH and NFL are not elevated in the plasma of SBMA patients [38, 39], this finding does not preclude their potential pathological importance. Genetic ablation of *Nefh* and the subsequent alteration of the 4:1:1 neurofilament subunit ratio (NFL:NFM:NFH) has been shown to alter the structural integrity of neurofilaments, resulting in decreased axon caliber and decreased neuronal conduction velocity [12, 28, 76]. It is known that the decreased uNFH observed in spinal motor neurons of the SBMA transgenic mouse model evaluated in this study is partially rescued upon disease amelioration, concomitant with a partial rescue of motor function, suggesting pathological importance [6, 50]. The decrease in uNFH seen in NMJs of the gastrocnemius provides evidence for a deficit at both ends of the motor neuron – in the soma [5] and at the NMJ. In contrast, NMJs of knock-in mouse TA show significantly increased pNFH. However, it should be noted that in both knock-in and transgenic mice, there is a subset of NMJs with higher pNFH intensity at the axon terminal. Previous studies have shown that proteasome dysregulation by genetic ablation of USP14 results in swellings of pNFH at the axon terminal [4]. Therefore, proteasome dysregulation, known to be present in SBMA [50, 59] might play a contributing role in the observed increased pNFH intensity at this subset of NMJs.

The NFH deficit seen in this study could result in decreased motor neuron conduction velocity, and therefore contribute to motor phenotypes. We considered carrying out electrophysiological studies to evaluate if motor neurons showing a decrease in NFH levels and/or distribution exhibited reduced conduction velocity, and to examine whether this correlated with glycolytic-to-oxidative fiber type switching seen in TA and gastrocnemius. However, stimulation and recording of the sciatic nerve would not allow for precise measurement of motor neurons innervating the muscles that have undergone fiber-type switching. To provide electrophysiological evidence for motor neurons innervating “switched” myofibers, motor neurons would need to be individually separated and evaluated for firing rate in vivo, which was beyond the scope of this study. Future studies using in vitro models of the neuromuscular junction could yield important information on motor neuron firing rate changes in SBMA models. In addition to its role in regulating axon diameter, NFH also has a role at the pre-synaptic terminal in determining the size of the readily releasable pool of synaptic vesicles and positioning organelles for energy needs [14, 76]. For example, NFH interacts with mitochondria, which are more abundant at the NMJ than at central synapses due to the high energy demands of the NMJ [68]. It is known that mitochondrial functions are altered in SBMA [52, 56]. Further experimentation to evaluate mitochondrial density at the NMJ due to neurofilament loss is warranted, as a decrease in mitochondrial density could contribute to neurotransmission failure in SBMA. Notably, loss of NFH could also be a downstream effect of neuronal dysfunction in the face of the toxic gain-of-function of the polyglutamine-expanded AR. Considering these data and the known roles of NFH in the neuron, the deficit in NFH localization at the pre-synaptic terminal could result in less readily available synaptic vesicles, fewer mitochondria, and a general lack of structural integrity, altering the functional ability of the nerve terminal. Future studies to elucidate the extent to which neurofilament proteins (NFL, NFM, NFH, and peripherin) are altered in SBMA mouse models in spinal cord motor neurons and sciatic nerve are needed to fully understand the role of neurofilaments in SBMA pathogenesis.

While fragmentation has canonically been used to describe disease pathology at the NMJ [10, 63], we observed significant NMJ fragmentation only in the TA muscle. The modest changes in fragmentation seen in this study, concomitant with significant changes in other measures of NMJ pathology, highlights the importance of including quantitative measurements when evaluating a disease such as SBMA, where neuronal and muscular alterations have a dynamic effect on the NMJ. While fragmentation was only significantly altered in TA, all three muscles presented with significant decreases in post-synaptic area and endplate area. These post-synaptic changes in soleus, TA, and gastrocnemius were accompanied by decreased muscle fiber cross-sectional area in all three muscles. It is known that post-synaptic size positively correlates with muscle fiber cross-sectional area [29, 61]. Therefore, the decrease in post-synaptic size could be secondary to the decrease in myofiber cross-sectional area or due to primary NMJ pathology. The directionality of this pathology is unable to be discerned from these studies. In addition, it is unclear whether the decrease in cross-sectional area is due to muscle atrophy or fiber type switching, as slow-twitch oxidative myofibers are smaller than fast-twitch glycolytic myofibers [3]. All three muscles evaluated showed a shift towards oxidative metabolism as measured by NADH-diaphorase staining, except for transgenic soleus muscle, which showed decreased NADH-diaphorase staining. The oxidative-to-glycolytic shift in transgenic soleus could be a result of altered metabolism, as mice deficient in mitochondrial long-chain fatty acid synthesis show an oxidative-to-glycolytic shift in slow-twitch soleus muscles [49]. The decreased NADH staining could also be due to reduced oxidative capacity, as mitochondrial depolarization and ATP production is decreased in models of SBMA [7, 52, 58]. It is worth noting that we cannot derive information on the efficiency of energy production in these muscles, as NADH-diaphorase staining measures only the presence of the necessary mitochondrial enzyme to deposit the stain, not mitochondrial activity, per se. Future metabolomic and proteomic studies on the muscles of these models will help to further deconstruct the observed metabolic changes.

It is important to clarify that while muscle fibers show convincing evidence of a glycolytic-to-oxidative shift in metabolic state based on NADH-diaphorase staining, these data do not support changes in myosin heavy chain isotype expression in gastrocnemius or TA that would indicate ‘fiber-type switching’ at a cytoskeletal protein expression level. The distinction between *metabolic* and *cytoskeletal* ‘fiber-type’ should be taken into consideration when evaluating neuromuscular disease, as the contractile properties of the myofiber may be discordant with its metabolic state. Previous studies have shown that mice defective in mitochondrial long chain fatty-acid synthesis showed alterations in mitochondrial metabolism that were discordant with myosin heavy chain expression [49]. Coordination of slow-twitch metabolic and cytoskeletal characteristics requires co-activation of PGC1α with Mef2 [35]. Mef2 transcriptional targets were shown to be decreased in knock-in mouse muscles due to sequestration of Mef2 in polyglutamine-expanded AR-containing intranuclear inclusions [47]. The evaluation of PGC1α protein levels in our study was inconclusive (data not shown). Nonetheless, it is likely that Mef2-specific transcriptional dysregulation [47], along with known global transcriptional and translational deficits [33, 62], contribute to the muscular cytoskeletal dysregulation observed in this study. Interestingly, increased vinculin protein expression observed in both mouse models of SBMA provides evidence for selective cytoskeletal changes that correlate with metabolic glycolytic-to-oxidative fiber type switching, as vinculin is known to be more highly expressed in slow-twitch oxidative myofibers [8]. Additionally, increased vinculin could be a sign of neurotransmission failure, as vinculin levels are known to increase upon denervation [57]. Understanding the nature of the distinct alteration in this myofiber cytoskeletal component is the focus of future studies. Of note, vinculin plays an analogous role in cardiac muscle. Vinculin’s role in SBMA cardiac phenotypes should be considered for further research, as SBMA patients have an increased prevalence of Brugada Syndrome, a cardiac syndrome that can lead to ventricular fibrillation and early death [1, 32].

In this study, we observed some differences between the two mouse models of SBMA. To understand the model-specific pathology, we considered that the mouse models used here differ in both age and polyglutamine-expanded AR expression. The transgenic model, which over-expresses polyglutamine-expanded AR primarily in the nervous system, showed no significant NMJ pathology at 6 months of age, despite significant motor deficits [5, 77]. In contrast, the knock-in model, which expresses endogenous polyglutamine-expanded AR, showed significant NMJ pathology in gastrocnemius muscle at 6 months of age. By 1-year of age, transgenic mice showed significant pathology in all three muscles evaluated. One possible explanation for the increased NMJ pathology in the transgenic mice is the higher levels of polyglutamine expanded AR in the spinal cord of the transgenic model [5, 77]. The overexpression of mutant AR in the spinal cord and brainstem could have contributed to broader NMJ pathology, independent of motor unit subtype, since mutant AR is overexpressed in all spinal cord neurons. The hypothesis that the differences between models are due to differences in polyglutamine expanded AR expression is not mutually exclusive of the hypothesis that the differences between models are due to the difference in age. The comparison of SBMA models of different ages is a limitation of this study; however this comparison evaluated NMJ pathology at timepoints known to be devoid of motor neuron loss. It is worth noting that primary spinal cord cultures from both the transgenic and knock-in mouse models exhibit substantial motor neuron cell death in response to hormone treatment [20, 45, 48, 50], indicating intrinsic motor neuron vulnerability in both models. Notably, it is unknown how polyglutamine-AR expression in glial cells of the spinal cord or terminal Schwann cells of the NMJ might affect the specificity of motor unit pathology. Further research into the role of glia in SBMA pathogenesis is needed, especially considering the importance of terminal Schwann cells in NMJ health.

A limitation of this study is the heterogenous nature of the muscles evaluated. While both gastrocnemius and TA represent “fast-twitch muscles”, they both contain a mixture of muscle fiber types that are known to change with age and anatomical location within the muscle [3, 25]. Because whole muscles were dissected and teased into small bundles prior to staining and imaging of *en face* NMJs, a random sample of NMJs was evaluated from each muscle. This random sample, combined with the heterogenous nature of the muscles evaluated, may have limited the power of this study to detect NMJ pathology in *specific* motor unit subtypes. However, while these data cannot provide insight on individual motor units, they do provide evidence to support increased vulnerability of muscles containing primarily fast-twitch myofibers in SBMA mouse models.

**Fig. 10:**
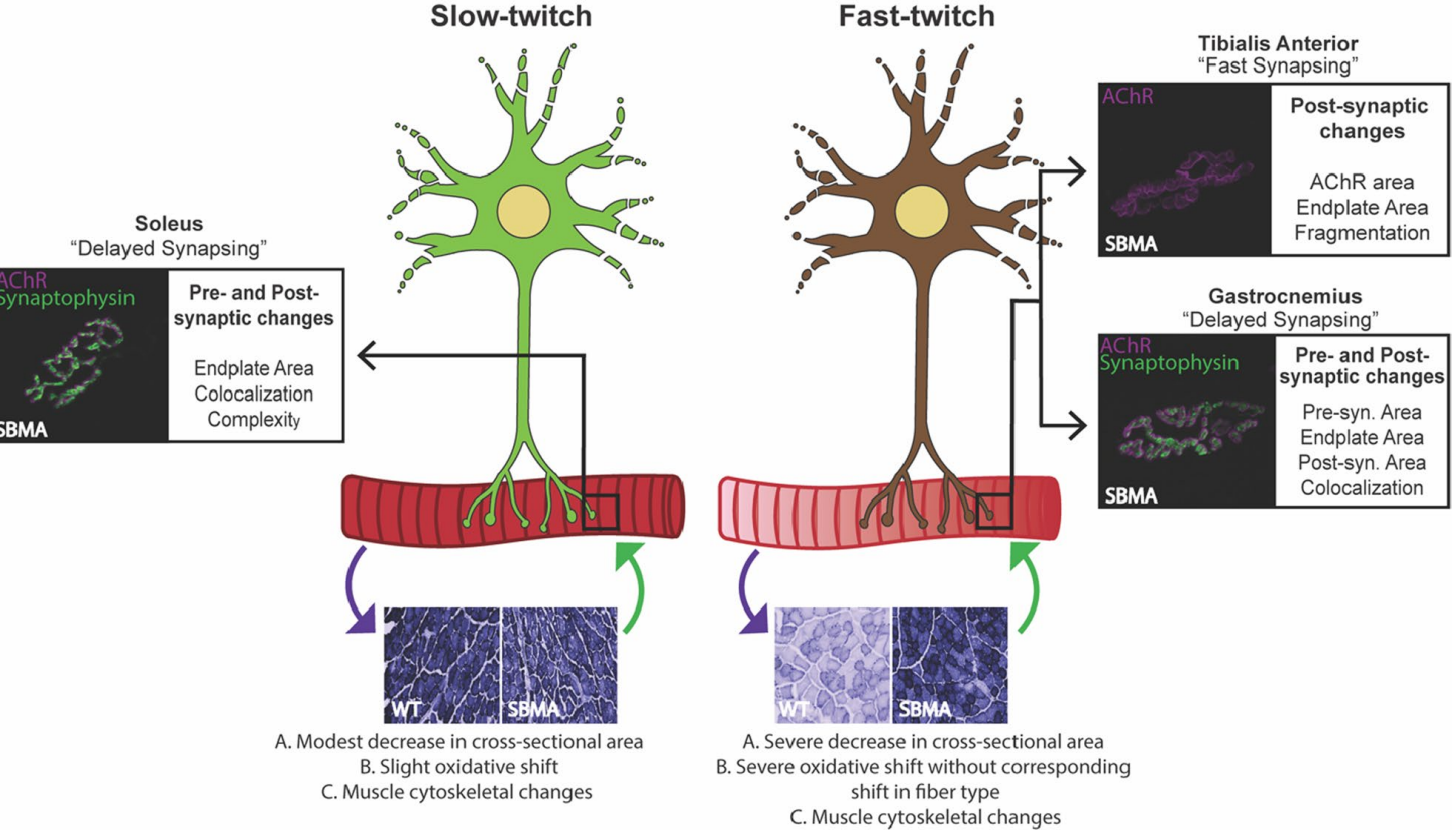
Model for NMJ and muscle pathology in SBMA mouse models. NMJ pathology reflects the contractile properties of the motor unit and metabolic machinery of the muscle fiber. We hypothesize that differences in the developmental subtype of the muscle also contributes to the response to disease. Slow-twitch soleus NMJs showed significant pre- and post-synaptic changes, with modest muscular pathology. Fast-twitch TA and gastrocnemius showed differing levels of NMJ pathology despite severe muscle atrophy and metabolic changes in both muscles. TA NMJs showed primarily post-synaptic pathology, while gastrocnemius showed significant pre- and post-synaptic NMJ pathology. We hypothesize the lower level of NMJ pathology in TA is due to the fast-synapsing (FaSyn) properties of TA during development, compared to the delayed-synapsing (DeSyn) properties of gastrocnemius. Together, the contractile properties of the motor unit and the developmental properties of the muscle fiber contribute to NMJ pathology in SBMA mouse models

Our model (Fig. 10) proposes that glycolytic-to-oxidative metabolic fiber-type alterations and decreased myofiber cross-sectional area are correlated with a decrease in NMJ post-synaptic membrane size and other NMJ pathologies, making fast-twitch motor units more vulnerable to SBMA pathogenesis than slow-twitch motor units. Glycolytic-to-oxidative motor unit changes and myofiber atrophy could be the result of either denervation, neuromuscular transmission dysfunction due to NMJ structural defects, or changes in motor neuron firing rate. Equally likely is that changes in myofiber metabolism and a decrease in fiber size promote changes in motor neuron firing rate and NMJ reorganization. Our quantitative analyses of NMJ pathology were largely in keeping with our hypothesis that NMJ pathology is correlated with muscle type vulnerability seen in SBMA mouse models and SBMA patients. Moreover, our study revealed a novel relationship between neurofilament dysregulation at the NMJ and both pre- and post-synaptic pathology. However, our observations of differences in NMJ pathology between gastrocnemius and TA suggest that motor unit vulnerability may also be impacted by inherent differences in the rate of synapse formation (e.g., fast synapsing vs. delayed synapsing). In addition, the oxidative shift in gastrocnemius and TA muscles, in the absence of myosin heavy chain isoform switching, may also contribute to the NMJ pathologies observed here. A deep understanding of the relationship between NMJ pathology and both neuronal and muscle alterations is likely to provide novel insights into approaches to enhance NMJ neurotransmission and disease progression in SBMA.

## Supplementary Information

**Additional file1**. Supplementary Methods. Supplementary Figs. 1–9. Supplementary Tables 1 and 2.

## Data Availability

All data generated or analyzed during this study are included in this published article [and its supplementary information files].

## Abbreviations

α-BTX: Alpha-bungarotoxin
AChR: Acetylcholine receptor
ALS: Amyotrophic lateral sclerosis
AR: Androgen receptor
CSA: Cross-sectional area
DeSyn: Delayed-synapsing
FaSyn: Fast-synapsing
KI: Knock-in
MHC: Myosin heavy chain
MIP: Maximum intensity projection
NADH: Nicotinamide adenine dinucleotide+hydrogen
NMJ: Neuromuscular junction
NTg: Non-transgenic
PFA: Paraformaldehyde
pNFH: Phosphorylated neurofilament heavy chain
SBMA: Spinal bulbar muscular atrophy
SMA: Spinal muscular atrophy
TA: Tibialis anterior
Tg: Transgenic
TUJ1: BIII-tubulin
uNFH: Unphosphorylated neurofilament heavy chain
WT: Wildtype
