# Supplementary material for: Neuromuscular junction pathology is correlated with differential motor unit vulnerability in spinal and bulbar muscular atrophy
Source: Acta Neuropathol Commun. 2022 Jul 5;10:97. doi: 10.1186/s40478-022-01402-y (PMC9258097; doi:10.1186/s40478-022-01402-y)
Supplement: Supplementary file 1 — Additional file1. Supplementary Methods. Supplementary Figs. 1–9. Supplementary Tables 1 and 2. [file 40478_2022_1402_MOESM1_ESM.pdf]

Supplementary Methods

Antibodies:

Primary antibodies used include AlexaFluor-647-conjugated a-bungarotoxin (ThermoFisher, 1:200; Waltham, MA), synaptophysin (ThermoFisher, 1:250; Waltham, MA), SMI31 (BioLegend, 1:1000; San Diego, CA), SMI32 (BioLegend, 1:1000; San Diego, CA), TUJ1 (Cell Signaling Technologies, 9F3, 1:500; Danvers, MA), Androgen Receptor (H280, 1:100), Vinculin (E1E9V, 1:1000; Cell Signaling Technologies; Danvers, MA), BA-D5 (Myosin heavy chain type I; 0.5mg/mL; DHSB; Iowa City, IA), BF-F3 (Myosin heavy chain type IB; 1:500; DHSB; Iowa City, IA), SC-71 (Mysoin heavy chain type IIa; 1.0 mg/mL; DHSB; Iowa City, IA). Secondary antibodies used include anti-Rabbit-488 (1:200; ThermoFisher; Waltham, MA), anti-Mouse IgG_1-594 (1:200; ThermoFisher; Waltham, MA), anti-rabbit-HRP (1:2000; Santa Cruz; Dallas, TX, anti-MsIgG2b-HRP (1:1000; Abcam; Cambridge, UK); anti-MsIgM-HRP (1:2000; ThermoFisher; Waltham, MA); anti-MsIgG1-HRP (1:1000; Abcam; Cambridge, UK); anti-MsIgG1-BP-HRP (1:3000; Santa Cruz; Dallas, TX).

Image Analysis of Neuromuscular Junctions

To analyze NMJs, we opened the image stack and created a maximum intensity projection (MIP) of each channel separately. Each MIP was then converted to an 8-bit image. **Post-synaptic area:** To evaluate post-synaptic area, threshold was set for α-bungarotoxin fluorescent image using Huang method of thresholding selected from the drop-down menu [22] or was set manually to accurately represent MIP. The thresholded image was then despeckled and made into a binary image. The area of the binary image was recorded. **Endplate area and AChR compactness:** To measure endplate area, we used *Process* → *subtract background* on the binary image. We then selected the binary image and recorded area measurement. The area output is the endplate area. To calculate acetylcholine receptor compactness, post-synaptic area was divided by endplate area. **Complexity:** From the binary image, the image was skeletonized. Using the point selection tool, the number of terminal branches for every endpoint in the skeletonized image was counted manually. This was recorded as "number of terminal branches". Using the point selection tool, the "number of branch points" was counted manually. Using the NMJ morph plug-in, we selected *Plugins* → *morphology* → *binary connectivity* then *analyze* → *histogram.* Histogram bins of 1-10 were totaled. This value represents the total branch length. We used the following equation to calculate complexity score: Complexity = $\log_{10}$(number terminal branches x number of branch points x total length of branches). **Pre-synaptic area:** To

evaluate pre-synaptic area, the protocol for evaluating post-synaptic area was repeated on synaptophysin fluorescent image. **Colocalization:** Using the binary images for pre-synaptic and post-synaptic areas described above and the thresholds recorded from pre- and post-synaptic area measurements, we used a colocalization plug-in to measure the number of colocalized points between the α-bungarotoxin fluorescent image and synaptophysin fluorescent image. **Neurofilament intensity:** Using binary images from the synaptophysin or α-bungarotoxin fluorescent images, we created a region of interest and restored that region of interest onto the fluorescent image of SMI31 or SMI32 stained NMJs; then intensity was measured as "mean gray value" and "integrated density". All images for intensity measurements were taken at a consistent exposure. **Neurofilament colocalization with terminal:** The colocalization plugin described above was also used to evaluated pNFH and uNFH localization to the terminal. For these data, pNFH localization to the terminal was measured as colocalization with the synaptophysin-stained pre-synaptic terminal, and uNFH localization to the terminal was measured as colocalization with α-bungarotoxin-stained post-synaptic terminal due to technical limitations.

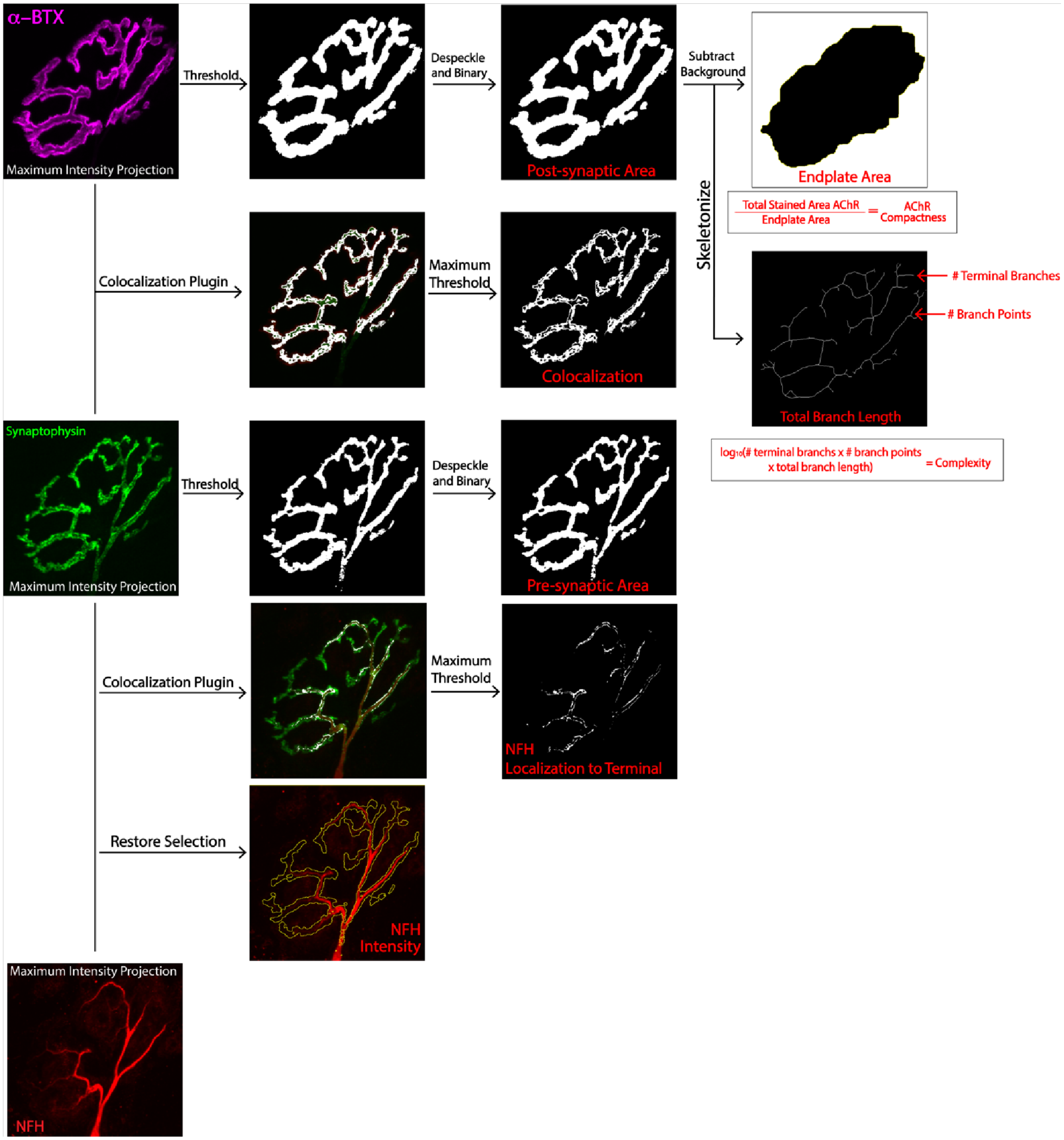

Supplementary Figure 1. Protocol for evaluating NMJ using ImageJ. Abbreviations: a-BTX, alpha-bungarotoxin; NFH, neurofilament heavy chain

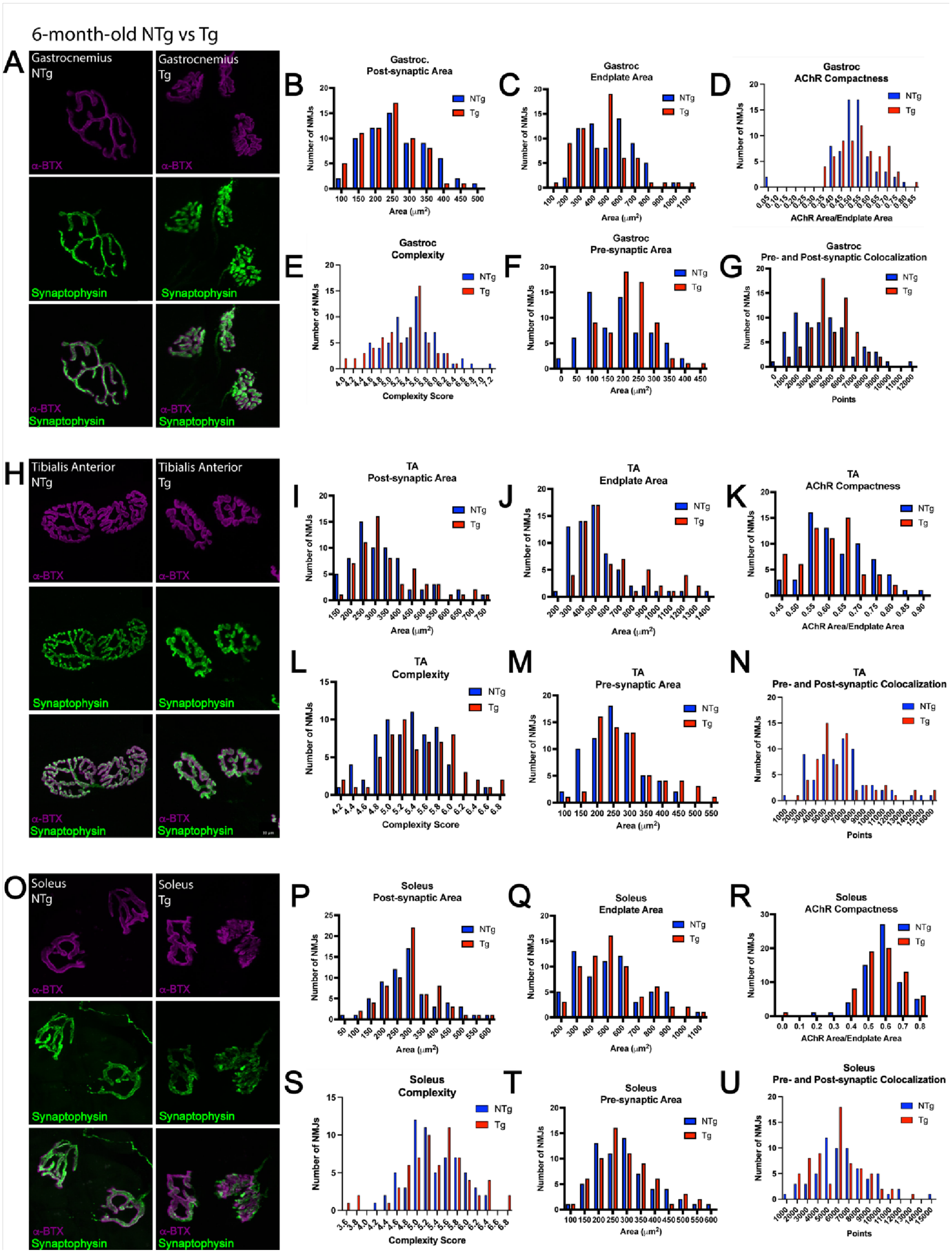
6-month-old NTg vs Tg
A
Gastrocnemius NTg
Gastrocnemius Tg
α-BTX
Synaptophysin
B
Gastroc. Post-synaptic Area
C
Gastroc Endplate Area
D
Gastroc AChR Compactness
AChR Area/Endplate Area
E
Gastroc Complexity
Complexity Score
F
Gastroc Pre-synaptic Area
G
Gastroc Pre- and Post-synaptic Colocalization
Points
H
Tibialis Anterior NTg
Tibialis Anterior Tg
I
TA Post-synaptic Area
J
TA Endplate Area
K
TA AChR Compactness
L
TA Complexity
M
TA Pre-synaptic Area
N
TA Pre- and Post-synaptic Colocalization
O
Soleus NTg
Soleus Tg
P
Soleus Post-synaptic Area
Q
Soleus Endplate Area
R
Soleus AChR Compactness
S
Soleus Complexity
T
Soleus Pre-synaptic Area
U
Soleus Pre- and Post-synaptic Colocalization
Number of NMJs
Area (μm²)
NTg
Tg

Supplementary Figure 2. 6-month-old transgenic mice show no significant NMJ pathology in gastrocnemius, tibialis anterior, and soleus muscles. Pre-synaptic membranes of NMJs from soleus muscle were stained with synaptic vesicle marker synaptophysin; post-synaptic membranes were labeled with fluorescently tagged a-bungarotoxin (a-BTX). NMJs from gastrocnemius, tibialis anterior, and soleus were evaluated for post-synaptic area, endplate area, AChR compactness, post-synaptic complexity, pre-synaptic area, and pre- and post-synaptic colocalization. No measurements showed significant changes at 6 months of age in any of the evaluated muscles. Mann-Whitney Test was used to evaluate statistical significance. Abbreviations: NTg, non-transgenic; Tg, transgenic; WT, wild-type; KI, knock-in

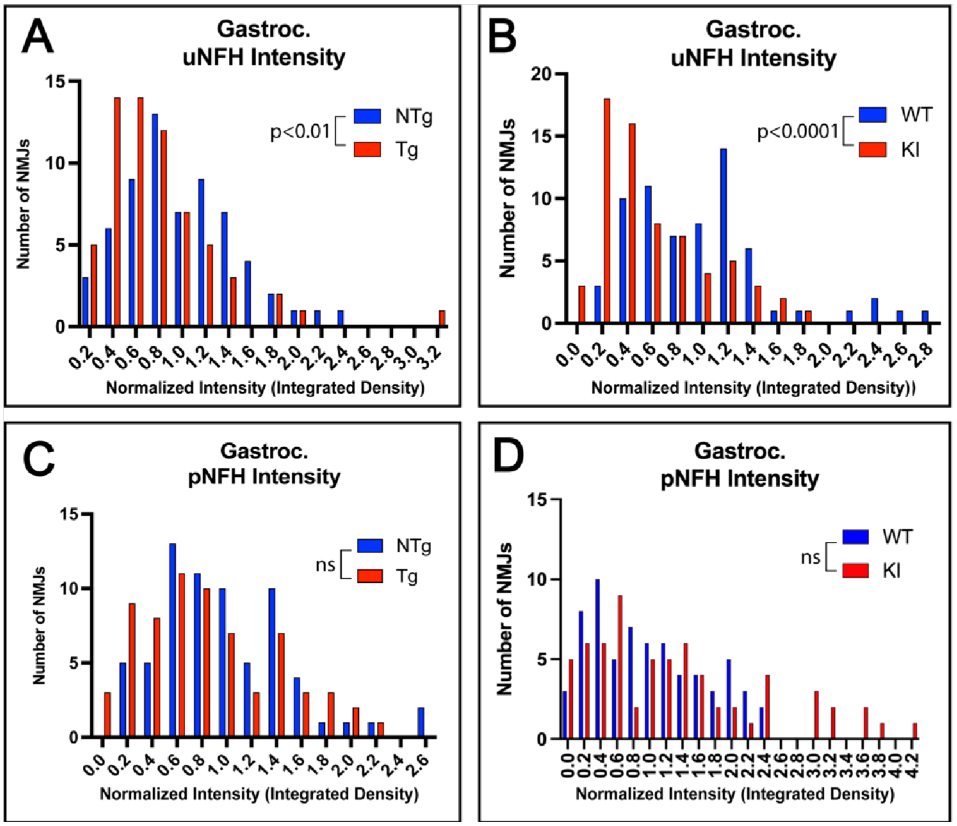

Supplementary Figure 3. NFH intensity is altered at the NMJ of gastrocnemius muscles in both transgenic and knock-in models of SBMA. To evaluate changes in cytoskeletal structural element NFH, NMJs from gastrocnemius were stained with a-BTX, synaptophysin, and SMI31 (phospho-NFH) or a-BTX, TUJ1 (BIII-tubulin, and SMI32 (unphospho-NFH) and evaluated for staining intensity. (A, B) Both transgenic ($p < 0.01$) and knock-in mice ($p < 0.0001$) showed significant decreases in the staining intensity of uNFH at the axon terminal compared to littermate controls. Neither mouse model showed a significant change in pNFH intensity at the axon terminal in gastrocnemius muscle, however transgenic NMJs (C) showed a trend towards a decrease. In contrast to transgenic mice, knock-in mice (D) showed a trend toward increased pNFH intensity at the axon terminal, although this did not reach significance. Mann-Whitney Test was used to evaluate statistical significance. Abbreviations: uNFH, unphosphorylated NFH; pNFH, phosphorylated NFH; NTg, non-transgenic; Tg, transgenic; WT, wild-type; KI, knock-in

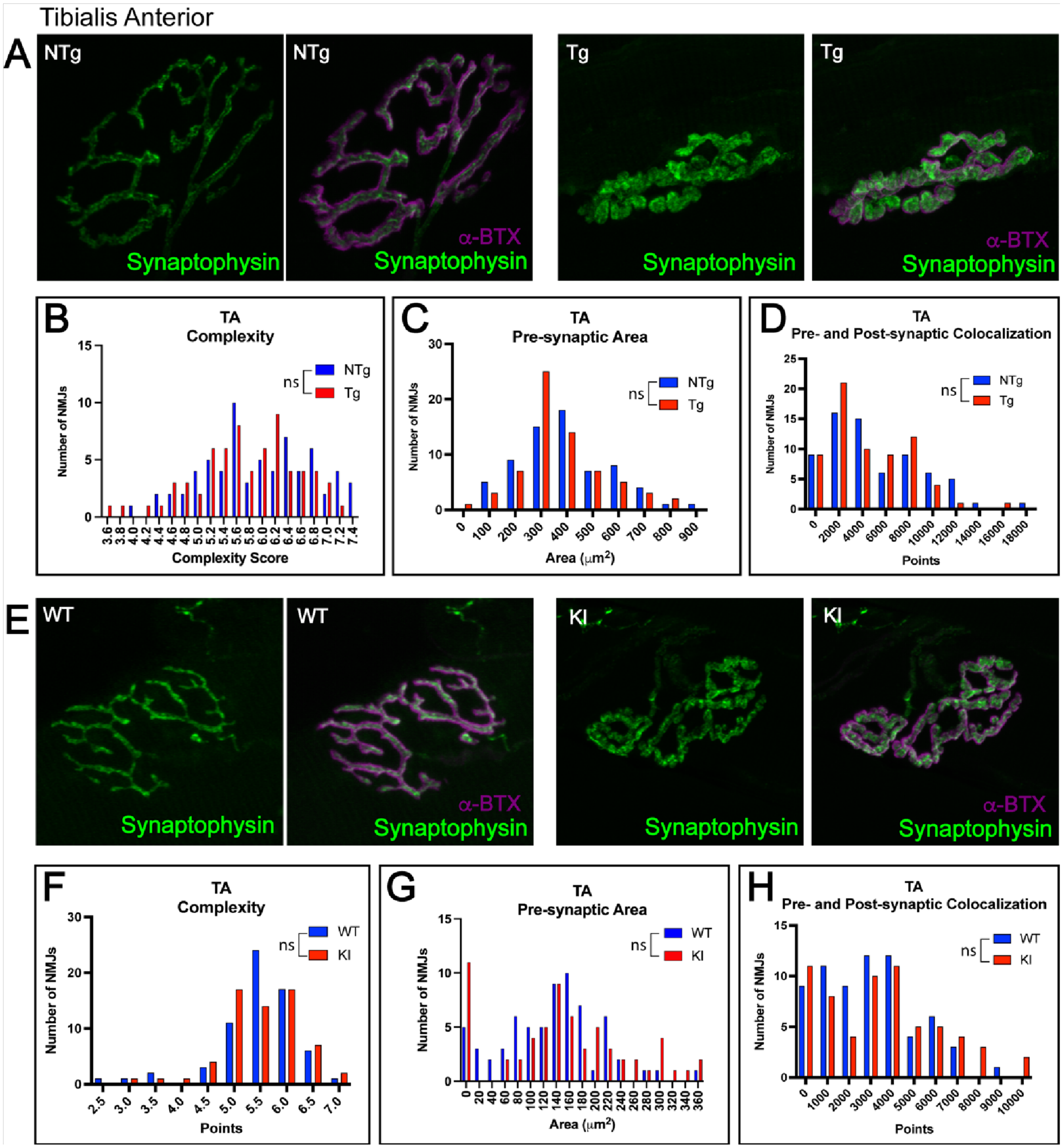

Supplementary Figure 4. Tibialis Anterior NMJs do not show significant changes in post-synaptic complexity, pre-synaptic area, or pre- and post-synaptic colocalization in either model of SBMA. Pre-synaptic terminals were stained with synaptic vesicle protein synaptophysin and measured for colocalization with the post-synaptic membrane labeled with fluorescently-tagged a-BTX. (A)Transgenic mouse NMJs were unaltered in measurements of (B) post-synaptic complexity, (C) pre-synaptic area, and (D) pre- and post-synaptic colocalization. (E) Knock-in mice NMJs were also unaltered in (F) post-synaptic complexity, (G) pre-synaptic area, and (H) pre- and post-synaptic colocalization. Mann-Whitney Test was used to evaluate statistical significance. Abbreviations: NTg, non-transgenic; Tg, transgenic; WT, wild-type; KI, knock-in

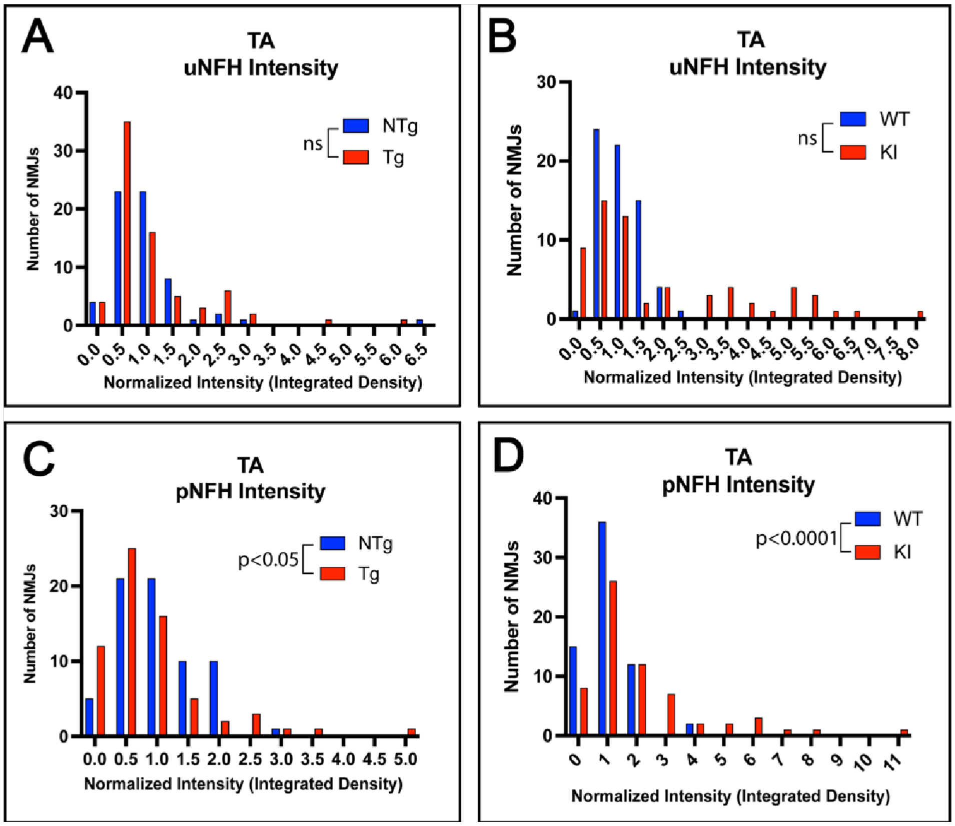

Supplementary Figure 5. pNFH staining intensity is altered at the NMJ of tibialis anterior muscles in both models of SBMA. To evaluate changes in cytoskeletal structural element NFH, NMJs from tibialis anterior were stained with a-BTX, synaptophysin, and SMI31 (phospho-NFH) or a-BTX, TUJ1 (BIII-tubulin), and SMI32 (unphospho-NFH) and evaluated for staining intensity. (A, B) In both knock-in and transgenic mice, changes in uNFH intensity did not reach statistical significance. However, (B) the distribution of NMJs in knock-in mice showed a distinct population of higher intensity NMJs that was not present in transgenic mice. (C, D) Both models showed a significant change in pNFH intensity but in opposite directions. Transgenic mice showed a *decrease* in pNFH intensity ($p < 0.05$), while knock-in mice showed an *increase* in pNFH intensity ($p < 0.0001$). As observed in B, a discrete population was present in the knock-in mice with higher intensity values. Mann-Whitney Test was used to evaluate statistical significance. Abbreviations: uNFH, unphosphorylated NFH; pNFH, phosphorylated NFH; NTg, non-transgenic; Tg, transgenic; WT, wild-type; KI, knock-in

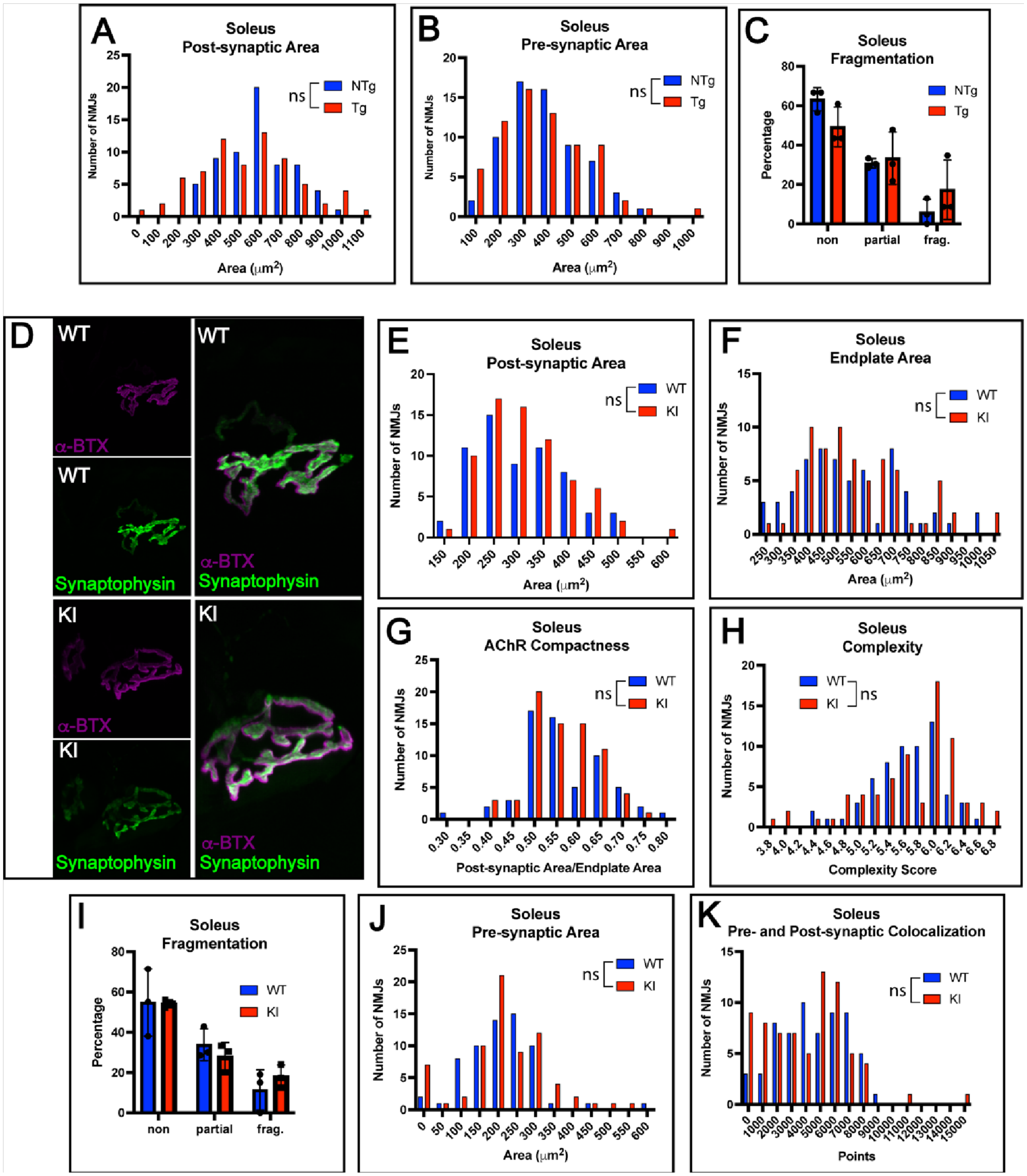

Supplementary Figure 6. Slow-twitch soleus does not show significant alterations in pre- or post-synaptic area or fragmentation in transgenic mice and does not show any NMJ pathology in knock-in mice. Pre-synaptic membranes of NMJs from soleus muscle were stained with synaptic vesicle marker synaptophysin, and post-synaptic membranes were labeled with fluorescently tagged a-BTX. Soleus NMJs in transgenic mice did not show significant changes in (A) post-synaptic area, (B) pre-synaptic area, or (C) NMJ fragmentation. (D) Knock-in mice did not show any significant change in NMJ pathology, including (E) post-synaptic area, (F) endplate area, (G) AChR compactness, (H) post-synaptic complexity, (I) NMJ fragmentation, (J) pre-synaptic area, or (K) pre- and post-synaptic colocalization. Mann-Whitney Test was used to

evaluate statistical significance. Abbreviations: NTg, non-transgenic; Tg, transgenic; WT, wild-type; KI, knock-in

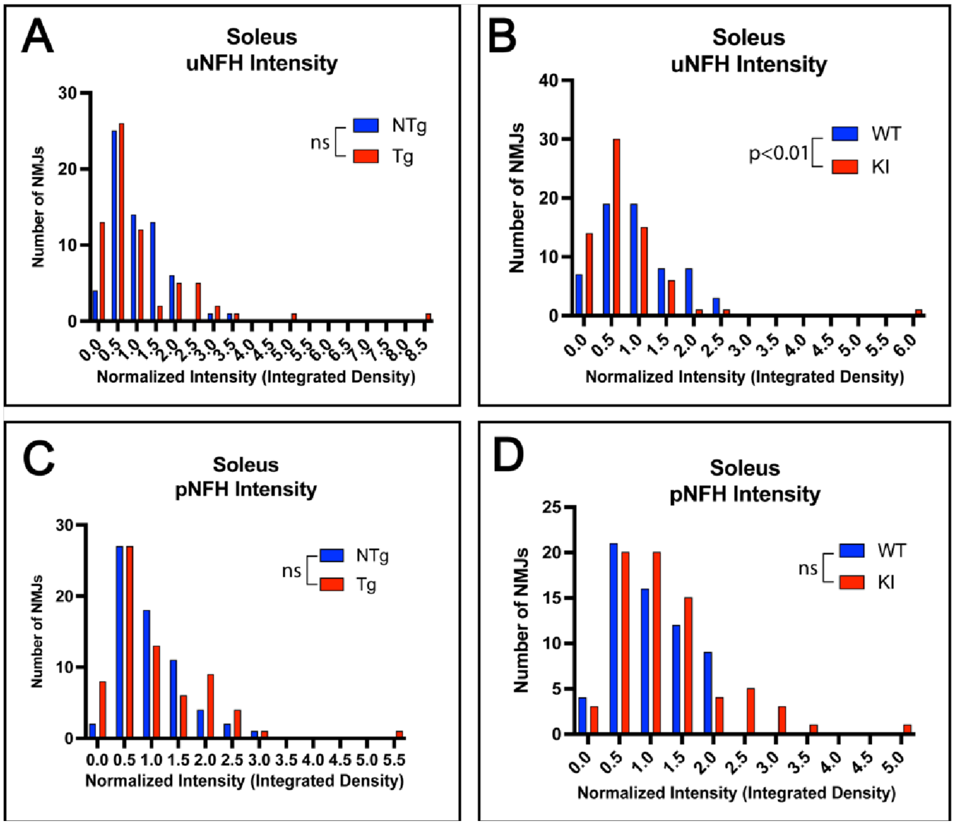

Supplementary Figure 7. uNFH intensity is altered at the NMJ of soleus muscles in knock-in mice, but not in transgenic mice. To evaluate changes in cytoskeletal structural element NFH, NMJs from gastrocnemius were stained with a-BTX, synaptophysin, and SMI31 (phospho-NFH) or a-BTX, TUJ1 (BIII-tubulin, and SMI32 (unphospho-NFH) and evaluated for staining intensity. (A) In transgenic soleus, NMJs did not show a significant change in uNFH intensity. (B) However, in knock-in soleus uNFH intensity was significantly decreased compared to wild-type littermates ($p < 0.01$). (C, D) In contrast, neither model showed a significant change in pNFH intensity, although there seems to be a subset of soleus NMJs in knock-in mice that shows higher levels of pNFH. Mann-Whitney Test was used to evaluate statistical significance. Abbreviations: uNFH, unphosphorylated neurofilament heavy chain; pNFH, phosphorylated neurofilament heavy chain; NTg, non-transgenic; Tg, transgenic; WT, wild-type; KI, knock-in

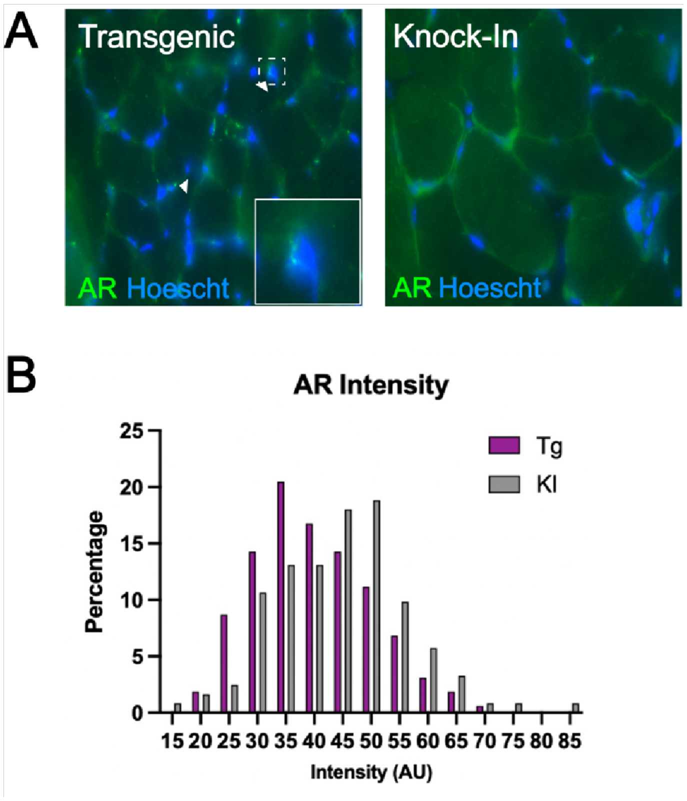

Supplementary Figure 8. Muscle androgen receptor expression is not significantly different between models. (A) Gastrocnemius muscle was immunostained for androgen receptor (AR) and evaluated for staining intensity. Arrowheads and inset show intranuclear inclusions. (B) There was no significant difference in AR staining intensity between transgenic and knock-in mouse models. Mann-Whitney Test was used to evaluate statistical significance. Abbreviations: AR, androgen receptor

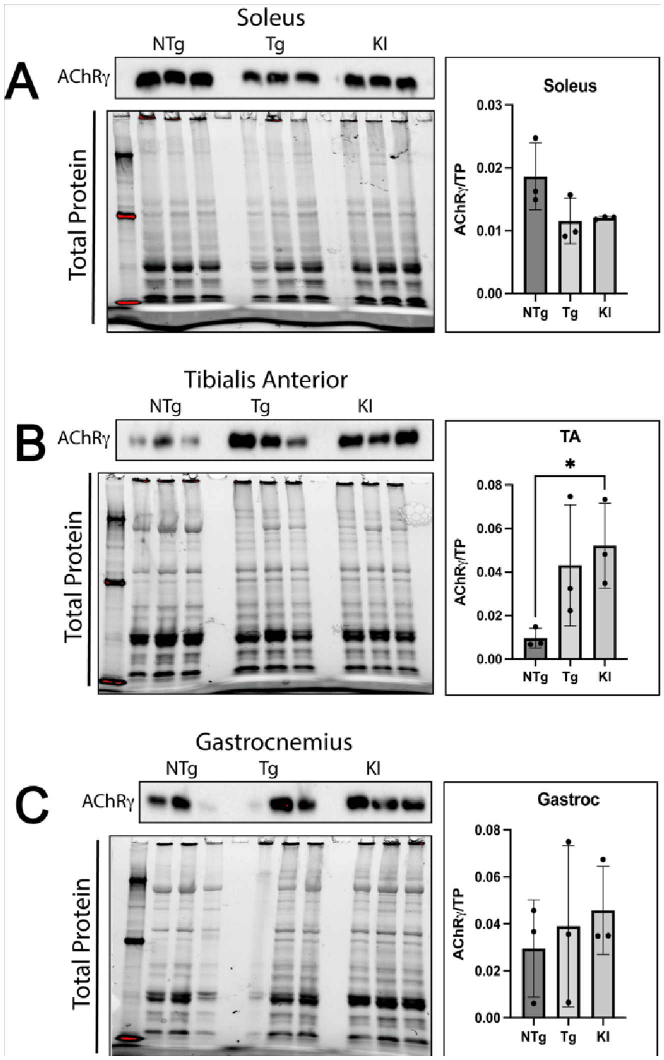

Supplementary Figure 9. AChR gamma subunit expression is increased in tibialis anterior but not soleus or gastrocnemius of SBMA mouse models. Western blots were probed for AChR-gamma subunit in 12-month-old transgenic and 9-month-old knock-in mouse soleus, tibialis anterior, and gastrocnemius muscle. (A) Soleus muscle showed a trend towards decreased AChR-gamma expression in both transgenic and knock-in mice. (B) Tibialis anterior showed elevated levels of AChR gamma in knock-in mice ($p < 0.05$) and trended higher in transgenic mice. (C) AChR gamma expression was unchanged in gastrocnemius in both transgenic and knock-in mice. Student's t-test was used to evaluate statistical significance. NTg = nontransgenic; Tg = transgenic; WT = wild-type; KI = knock-in

Table 1

	Non-Transgenic vs Transgenic							Wildtype vs Knock-In					
**Muscle**	**Measurement**	**p-value**	**NTg Median**	**Tg Median**	**NTg Mean +/- SD**	**Tg Mean +/- SD**		**Measurement**	**p-value**	**NTg Median**	**Tg Median**	**NTg Mean +/- SD**	**Tg Mean +/- SD**
Gastrocnemius	Post-synaptic area	<0.0001	606.3	419.4	597.6 ± 191.5	455.9 ± 175.5		Post-synaptic area	<0.0001	244.5	187.9	248.3 ± 62.63	199.2 ± 60.98
	Endplate Area	<0.0001	1138	842.9	1207 ± 464.3	858.9 ± 327.2		Endplate Area	<0.0001	514.3	371.8	522.6 ± 159.9	385.8 ± 155.5
	AChR Compactness	0.0551	0.4927	0.5465	0.5126 ± 0.08402	0.5386 ± 01046		AChR Compactness	0.0009	0.481	0.5517	0.4883 ± 0.07549	0.5422 ± 0.1004
	Fragmentation	0.2715			16.85 ± 14.95	31.91 ± 13.98		Fragmentation	0.2966			3.037 ± 2.638	11.8 ± 12.37
	Pre-synaptic area	<0.0001	386.5	304.8	394.9 ± 140.3	290.4±119.2		Pre-synaptic area	<0.0001	163.1	117.1	157.7 ± 58.45	112.2 ± 71.15
	Pre- and Postsynaptic Colocalization	0.0009	7747	4671	7945 ± 4545	5359 ± 3528		Pre- and Postsynaptic Colocalization	0.0028	3433	2264	3078 ± 2073	2099 ± 1523
	Complexity	0.0586	6.45	6.247	6.283 ± 0.8482	6.066 ± 0.6842		Complexity	0.0112	5.51	5.271	5.446 ± 0.4782	5.169 ± 0.6335
	pNFH Intensity	0.0672	0.9582	0.7551	1.00 ± 0.5289	0.8375 ± 0.5526		pNFH Intensity	0.2571	0.9002	1.114	1.00 ± 0.6690	1.309 ± 1.065
	pNFH Colocalization with Terminal	0.0007	1197	459	1289 ± 1051	744.1 ± 811.3		pNFH Colocalization with Terminal	0.0017	882	516	938.7 ± 621.1	669.8 ± 761.3
	uNFH Intensity	0.0054	0.9085	0.6899	1.00 ± 0.4767	0.805 ± 0.5043		uNFH Intensity	<0.0001	0.9874	0.4197	1.00 ± 0.5713	0.5861 ± 0.4488
	uNFH Colocalization with Terminal	0.0618	1194	833	1289 ± 936.6	951.4 ± 613.3		uNFH Colocalization with Terminal	<0.0001	1289	614	1262 ± 631.1	676.2 ± 565.9
	**Non-Transgenic vs Transgenic**							**Wildtype vs Knock-In**					
**Muscle**	**Measurement**	**p-value**	**NTg Median**	**Tg Median**	**NTg Mean +/- SD**	**Tg Mean +/- SD**		**Measurement**	**p-value**	**WT Median**	**KI Median**	**WT Mean +/- SD**	**KI Mean +/- SD**
Tibialis Anterior	Post-synaptic area	0.0011	647	523.6	713.5 ± 311.3	543 ± 193		Post-synaptic area	0.3604	263.4	272.8	273.8 ± 124.8	294.2 ± 114.1

	Endplate Area	0.0023	1229	1022	1461 ± 737	1079 ± 442.1		Endplate Area	0.9012	536.4	539.5	567.6 260.7	552.6 ± 226.1
	AChR Compactness	0.5492	0.5092	0.5266	0.5126 ± 0.09471	0.5223 ± 0.09330		AChR Compactness	0.0227	0.5031	0.5309	0.5112 ± 0.09690	0.5441 ± 0.08749
	Fragmentation	0.0248			8.799 ± 4.491	25.36 ± 6.847		Fragmentation	0.4369			10.57 ± 3.240	12.72 ± 2.851
	Pre-synaptic area	0.4703	377.7	339.1	393 ± 172.9	372.1 ± 159.3		Pre-synaptic area	0.3615	139.6	141.5	133.7 ± 75.04	151.8 ± 99.16
	Pre- and Postsynaptic Colocalization	0.3577	4286	3539	5326 ± 4004	4610 ± 3396		Pre- and Postsynaptic Colocalization	0.6456	2957	3271	3016 ± 2109	3398 ± 2668
	Complexity	0.2228	5.95	5.743	5.948 ± 0.8288	5.743 ± 0.7824		Complexity	0.9833	5.605	5.591	5.479 ± 0.7852	5.597 ± 0.7186
	pNFH Intensity	0.034	0.8486	0.6557	1 ± 0.5962	0.8975 ± 0.8819		pNFH Intensity	<0.0001	0.8016	1.404	1.00 ± 0.7369	2.178 ± 2.105
	pNFH Colocalization with Terminal	<0.0001	1081	445	1328 ± 1033	725.4 ± 872.9		pNFH Colocalization with Terminal	0.6324	974	745.5	995.5 ± 766.4	1068 ± 1164
	uNFH Intensity	0.4853	0.8968	0.7096	1 ± 0.8763	1.059 ± 1.007		uNFH Intensity	0.167	1.055	1.081	1.00 ± 0.4992	2.008 ± 1.995
	uNFH Colocalization with Terminal	<0.0001	1102	407	1348 ± 1115	747.7 ± 815.1		uNFH Colocalization with Terminal	0.8318	1234	1197	1232 ± 711.2	1276 ± 714.7
	**Non-Transgenic vs Transgenic**								**Wildtype vs Knock-In**				
**Muscle**	**Measurement**	**p-value**	**NTg Median**	**Tg Median**	**NTg Mean +/- SD**	**Tg Mean +/- SD**		**Measurement**	**p-value**	**WT Median**	**KI Median**	**WT Mean +/- SD**	**KI Mean +/- SD**
Soleus	Post-synaptic area	0.0720	593	523	594.7 ± 170.3	531.0 ± 240.1		Post-synaptic area	0.5623	286.3	296.9	303.2 ± 91.88	310.8 ± 87.32
	Endplate Area	0.0152	1098	889	1157 ± 419.5	1007 ± 530.3		Endplate Area	0.7137	516.1	519.8	547.3 ± 180.6	561.8 ± 177.2
	AChR Compactness	0.0566	5430	5647	0.5348 ± 0.09320	0.5591 ± 0.08923		AChR Compactness	0.9168	0.5411	0.5537	0.5667 ± 0.09235	0.5637 ± 0.07649
	Fragmentation	0.294			5.935 ± 6.60	17.39 ± 15.06		Fragmentation	0.3784			11.35 ± 10.03	17.96 ± 5.772
	Pre-synaptic area	0.3368	369.8	352.2	394.1 ± 149.6	376.3 ± 186.7		Pre-synaptic area	0.8834	216	203.3	212.0 ± 94.96	214.4 ± 112.4
	Pre- and Postsynaptic Colocalization	0.0103	7182	5058	7605 ± 3993	5881 ± 4168		Pre- and Postsynaptic Colocalization	0.1613	4441	4513	4581 ± 2233	4078 ± 2855

	Complexity	0.0001	6.414	5.826	6.239 ± 0.7097	5.793 ± 0.8272		Complexity	0.3603	5.722	5.916	5.661 ± 0.4695	5.693 ± 0.6406
	pNFH Intensity	0.8279	0.8946	0.7454	1.00 ± 0.6190	1.055 ± 0.8866		pNFH Intensity	0.1202	0.9028	1.061	1.00 ± 0.4991	1.04 ± 0.5535
	pNFH Colocalization with Terminal	0.007	1750	997	2118 ± 1655	1475 ± 1452		pNFH Colocalization with Terminal	0.3604	1483	1302	1593 ± 886.5	1453 ± 997.9
	uNFH Intensity	0.1661	0.7908	0.6017	1.00 ± 0.6644	1.091 ± 1.311		uNFH Intensity	0.0013	0.89	0.5154	1.00 ± 0.6460	0.7250 ± 0.8308
	uNFH Colocalization with Terminal	0.4837	1678	1370	2161 ± 1826	2257 ± 3131		uNFH Colocalization with Terminal	0.0175	1365	871.5	1530 ± 1009	1236 ± 1212

Table 1: Data for all measurements of NMJ pathology for 3 mice per genotype, $\geq$ 20 NMJs evaluated per muscle, per genotype. pvalue for Mann-Whitney test of NTg vs Tg and WT vs Knock-In.

Table 2

	Non-Transgenic vs Transgenic						
**Muscle**	**Measurement**	**p-value**	**NTg Median**	**Tg Median**	**NTg Mean +/- SD**	**Tg Mean +/- SD**	
Gastrocnemius	NADH Staining Intensity	< 0.0001	0.006453	0.009022	0.007639 +/- 0.003109	0.01006 +/- 0.003046	
	Cross-sectional Area	< 0.0001	2328	1156	2398 +/- 737.1	1235 +/- 434.1	
Tibialis Anterior	NADH Staining Intensity	< 0.0001	0.004336	0.00354	0.01093 +/- 0.006430	0.01398 +/- 0.004846	
	Cross-sectional Area	< 0.0001	2442	1026	2467 +/- 644.9	1074 +/- 338.6	
Soleus	NADH Staining Intensity	< 0.0001	0.0162	0.1107	0.01653 +/- 0.004963	0.0122 +/- 0.005496	
	Cross-sectional Area	< 0.0001	1777	932	1939 +/- 724.6	1063 +/- 585.1	

	Wildtype vs Knock-In						
Muscle	Measurement	p-value	WT Median	KI Median	WT Mean +/- SD	KI Mean +/- SD	
Gastrocnemius	NADH Staining Intensity	0.0001	0.007924	0.008763	0.01017 +/- 0.004748	0.009834 +/- 0.003569	
	Cross-sectional Area	< 0.0001	2218	1136	2219 +/- 867	1232 +/- 473	
Tibialis Anterior	NADH Staining Intensity	<0.0001	0.008286	0.01082	0.01043 +/- 0.005096	0.01279 +/- 0.005938	
	Cross-sectional Area	<0.0001	2196	1249	2197 +/- 798.2	1305 +/- 379.4	
Soleus	NADH Staining Intensity	< 0.0001	0.01697	0.01846	0.0163 +/- 0.005073	0.01991 +/- 0.006589	
	Cross-sectional Area	0.0013	1512	1431	1621 +/- 584.8	1531 +/- 544.4	

Table 2: Data for cross-sectional area and NADH staining intensity (1/FIJI arbitrary intensity units). P-value for Mann-Whitney test of NTg vs Tg and WT vs Knock-in.
